# Mutational and structural studies of (βα)_8_‐barrel fold methylene‐tetrahydropterin reductases utilizing a common catalytic mechanism

**DOI:** 10.1002/pro.5018

**Published:** 2024-05-15

**Authors:** Manuel Gehl, Ulrike Demmer, Ulrich Ermler, Seigo Shima

**Affiliations:** ^1^ Max Planck Institute for Terrestrial Microbiology Marburg Germany; ^2^ Max Planck Institute of Biophysics Frankfurt am Main Germany

**Keywords:** catalytic mechanism, crystal structure, evolution, methylene‐tetrahydrofolate reductase, methylene‐tetrahydromethanopterin reductase

## Abstract

Methylene‐tetrahydropterin reductases catalyze the reduction of a methylene to a methyl group bound to a reduced pterin as C_1_ carrier in various one‐carbon (C_1_) metabolisms. F_420_‐dependent methylene‐tetrahydromethanopterin (methylene‐H_4_MPT) reductase (Mer) and the flavin‐independent methylene‐tetrahydrofolate (methylene‐H_4_F) reductase (Mfr) use a ternary complex mechanism for the direct transfer of a hydride from F_420_H_2_ and NAD(P)H to the respective methylene group, whereas FAD‐dependent methylene‐H_4_F reductase (MTHFR) uses FAD as prosthetic group and a ping–pong mechanism to catalyze the reduction of methylene‐H_4_F. A ternary complex structure and a thereof derived catalytic mechanism of MTHFR is available, while no ternary complex structures of Mfr or Mer are reported. Here, Mer from *Methanocaldococcus jannaschii* (jMer) was heterologously produced and the crystal structures of the enzyme with and without F_420_ were determined. A ternary complex of jMer was modeled on the basis of the jMer‐F_420_ structure and the ternary complex structure of MTHFR by superimposing the polypeptide after fixing hydride‐transferring atoms of the flavins on each other, and by the subsequent transfer of the methyl‐tetrahydropterin from MTHFR to jMer. Mutational analysis of four functional amino acids, which are similarly positioned in the three reductase structures, indicated despite the insignificant sequence identity, a common catalytic mechanism with a 5‐iminium cation of methylene‐tetrahydropterin as intermediate protonated by a shared glutamate. According to structural, mutational and phylogenetic analysis, the evolution of the three reductases most likely proceeds via a convergent development although a divergent scenario requiring drastic structural changes of the common ancestor cannot be completely ruled out.

## 1 INTRODUCTION

Redox reactions of C_1_ units bound to C_1_ carriers are widespread in the three domains of life. The most common C_1_ carriers are tetrahydrofolate (H_4_F) and tetrahydromethanopterin (H_4_MPT), which consist of a reduced pterin (tetrahydropterin) bound to a *para*‐aminobenzoate (PABA) group or its para‐aniline derivative, respectively, and a variable tail region (Maden, 2000). H_4_F and H_4_MPT significantly differ (Figure S1), as H_4_MPT has a methylene group and H_4_F has a carbonyl group both conjugated to N10 via an aromatic ring. The structural differences are reflected in different p*K*_a_ values for N10 and in substantially different redox properties of the C_1_ unit attached (Thauer et al., 1996). The bound C_1_ unit is transformed between oxidation states of formic acid (+II, formyl and methenyl group), formaldehyde (0, methylene group) and methanol (−II, methyl group) in the C_1_ metabolisms. H_4_F is employed in the methyl‐branch of the Wood–Ljungdahl pathway, and the folate cycle of most organisms, while H_4_MPT is utilized in the catabolic pathways of methanogenic and sulfate‐reducing archaea, as well as methylotrophic and methanotrophic bacteria (Borrel et al., 2016; Zheng & Cantley, 2019).

**FIGURE 1:**
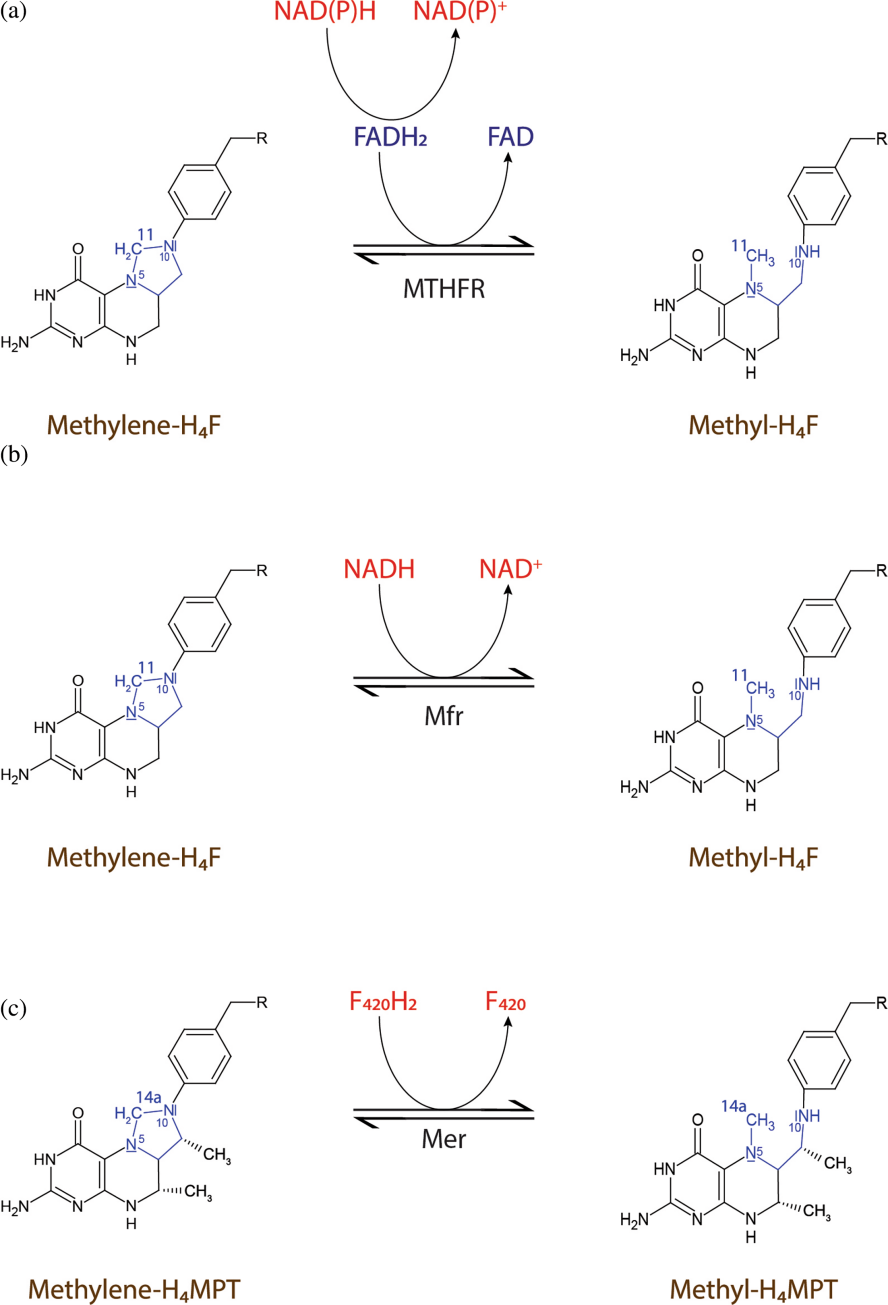
Reactions catalyzed by methylene‐tetrahydropterin reductases. (a) FAD‐dependent methylene‐H_4_F reductases (MTHFR) catalyzes the reduction of methylene‐H_4_F by NAD(P)H using a ping–pong mechanism with FAD, bound to the enzyme as a prosthetic group, as intermediate redox center. (b) Flavin‐independent methylene‐H_4_F reductase (Mfr) catalyzes the reduction of methylene‐H_4_F by using NADH as reducing agent. In contrast to MTHFR, Mfr has no prosthetic group and uses a ternary complex reaction mechanism. (c) F_420_‐dependent methylene‐H_4_MPT reductase (Mer) catalyzes the reduction of methylene‐H_4_MPT with reduced F_420_ (F_420_H_2_) as reducing agent via a ternary complex mechanism.

The reduction of methylene‐H_4_F to methyl‐H_4_F is catalyzed by methylene‐H_4_F reductases (Sah et al., 2020; Sheppard et al., 1999; Zheng & Cantley, 2019), which are classified into a flavin‐dependent enzyme (MTHFR) and a flavin‐independent one (Mfr) (Sah et al., 2020; Yu et al., 2022). MTHFR is further classified into subclasses depending on either FAD or FMN as a prosthetic group (Bertsch et al., 2015; Clark & Ljungdahl, 1984; Mock et al., 2014; Öppinger et al., 2022; Sheppard et al., 1999). The FAD‐dependent MTHFR uses a ping–pong reaction mechanism (Figure 1a), in which the tightly bound FAD is first reduced by NAD(P)H and then FADH_2_ reduces methylene‐H_4_F (Trimmer et al., 2001). The structural characterization of the inactive MTHFR_Glu28Gln variant from *E. coli* (eMTHFR) with FAD and with either NADH or methyl‐H_4_F (Pejchal et al., 2005) revealed both substrates in the same position, explaining the structural basis for the ping–pong mechanism. Since the imidazolidine ring in methylene‐H_4_F is a poor acceptor for the negatively charged hydride, the substrate is activated to a 5‐iminium cation (Sumner & Matthews, 1992). A 5‐iminium cation intermediate was also proposed in the non‐enzymatic condensation of formaldehyde and H_4_F to form methylene‐H_4_F (Kallen & Jencks, 1966). Additionally, in the crystal structure of thymidylate synthase, a 5‐hydroxymethylene‐H_4_F was found, which supports the presence of the 5‐iminium cation as an intermediate in this enzyme reaction (Perry et al., 1993). Based on the drastic effect of the Glu28Gln mutation of eMTHFR on the activity, Glu28 was proposed to be the catalytic acid for protonating the substrate for the formation of the 5‐iminium cation.

Recently, the enzymes encoded by *MSMEG_6596* and *MSMEG_6649* in *Mycobacterium smegmatis* were biochemically characterized and MTHFR activity demonstrated (Sah et al., 2020). Both enzymes do not contain flavin and catalyze a direct hydride transfer from NADH to methylene‐H_4_F by a ternary complex mechanism (Figure 1b). The flavin‐independent enzymes, named Mfr (Gehl et al., 2023), was currently identified only in mycobacteria as a monomeric enzyme. A knockout strain of *M. smegmatis* showed impaired growth in the absence of methionine, suggesting that Mfr is involved in the methionine cycle in mycobacteria (Sah et al., 2020). Likewise, an Mfr homolog encoded by *Rv2172c* in *M. tuberculosis* is essential for the growth of this organism (Yu et al., 2022). Recently, we reported the crystal structure of Mfr from *Mycolicibacterium hassiacum* (hMfr) and provided evidence that Glu9 in the active site is the key catalytic residue for 5‐iminium cation formation. Thus, the mechanism of eMTHFR and hMfr is basically identical.

Most methanogenesis pathways involve the methylene‐H_4_MPT reductase (Mer), which catalyzes the reversible reduction of methylene‐H_4_MPT with F_420_H_2_ as reductant using a ternary complex mechanism (Kurth et al., 2020; Ma & Thauer, 1990a) (Figure 1c). The product methyl‐H_4_MPT is an intermediate of the energy metabolism and the methyl donor of acetyl‐coenzyme A biosynthesis. Crystal structures are available for substrate‐free Mer from *Methanopyrus kandleri* and *Methanothermobacter marburgensis* (Shima et al., 2000) and the *Methanosarcina barkeri* Mer‐F_420_ binary complex (Aufhammer et al., 2005). Mer belongs to the bacterial luciferase family, which consists of FMN‐ and F_420_‐dependent oxidoreductases (Aufhammer et al., 2005).

In this report, the hydride‐transfer mechanism of three functionally related methylene‐tetrahydropterin reductases was comparatively scrutinized with the aim to better understand their phylogenetic relationship in terms of divergent and convergent evolution. In the divergent scenario, the three hydride‐transferring enzymes would originate from a common ancestor, while in a convergent scenario, they start from a different origin and develop under evolutionary pressure to shared active site features. For the analysis, Mer from *Methanocaldococcus jannaschii* was chosen as only the enzyme from this organism can be heterologously and functionally produced thus allowing mutational analyses. For structural comparison with eMTHFR and hMfr, the crystal structures of Mer from *M. jannaschii* (jMer) with and without F_420_ were solved. We identified similar amino acid residues at equivalent positions in the active sites of eMTHFR, hMfr, and jMer, which are potentially involved in the binding and activation of the C_1_ carriers. Based on mutational and kinetic analysis, evidence has been provided that all three methylene‐tetrahydropterin reductases have the same fold, similar methylene‐tetrahydropterin binding positions, and the same basic catalytic mechanism; however, the binding modes of the C_1_ carriers and reductants significantly deviate. In addition, we obtained the crystal structures of the inactive variants jMer_E6Q, and hMfr_E9Q to exclude perturbations of the active sites.

## 2 RESULTS AND DISCUSSION

### 2.1 Characterization of the heterologously produced jMer

Attempts to produce Mer from different organisms in *E. coli* have failed in the past because Mer formed inclusion bodies (Vaupel & Thauer, 1995). However, recently, the first successful heterologous expression of Mer from *Methanocaldococcus jannaschii* (jMer) was reported, in which the heterologously produced jMer catalyzed the formation of lactaldehyde by reduction of methylglyoxal with NADPH (Miller et al., 2017). We obtained the published expression system from the authors and characterized the purified enzyme. The apparent *K*_m_ and *k*_cat_ values of jMer are in the range of those of the respective enzymes from methanogenic archaea (*M. barkeri*, *M. marburgensis*, and *M. kandleri*) and sulfate‐reducing archaea (*Archaeoglobus fulgidus*) (Table 1) (Ma et al., 1991; Ma & Thauer, 1990a; Ma & Thauer, 1990b; Schmitz et al., 1991; te Brommelstroet et al., 1990). Size‐exclusion chromatography showed that jMer in solution is a homodimer (~80 kDa) (Figures S2 and S3) as reported in the literature (Miller et al., 2017).

**TABLE 1:** Comparison of the kinetic constants of Mer from *Methanosarcina barkeri*, *Methanothermobacter marburgensis*, *Methanopyrus kandleri*, *Archaeoglobus fulgidus*, *Methanocaldococcus jannaschii*.

Mer from	Temperature^a^		Apparent *K*_m_	Apparent *k*_cat_	Source
*M. barkeri*	37°C (55°C)	Methylene‐H_4_MPT	15 μM	76,000 min^−1^ (1300 s^−1^)	(Ma & Thauer, 1990b)
		F_420_H_2_	12 μM		
*M. marburgensis*	65°C (55°C)	Methylene‐H_4_MPT	300 μM	220,000 min^−1^ (3600 s^−1^)	(Ma & Thauer, 1990a)
		F_420_H_2_	3 μM		
*M. kandleri*	98°C (65°C)	Methylene‐H_4_MPT	7 μM	16,000 min^−1^ (270 s^−1^)	(Ma et al., 1991)
		F_420_H_2_	4 μM		
*A. fulgidus*	83°C (65°C)	Methylene‐H_4_MPT	16 μM	17,000 min^−1^ (280 s^−1^)	(Schmitz et al., 1991)
		F_420_H_2_	4 μM		
*M. jannaschii*	85°C (55°C)	Methylene‐H_4_MPT	58 μM	18,000 min^−1^ (300 s^−1^)	This work
		F_420_H_2_	5 μM		

*Note*: Mer from *M. jannaschii* was the only tested enzyme heterologously produced in *Escherichia coli*.

^a^ The temperatures indicate the growth optimum temperature. The enzyme assay temperature was given in parenthesis.

### 2.2 Determination of the crystal structure of jMer

**FIGURE 2:**
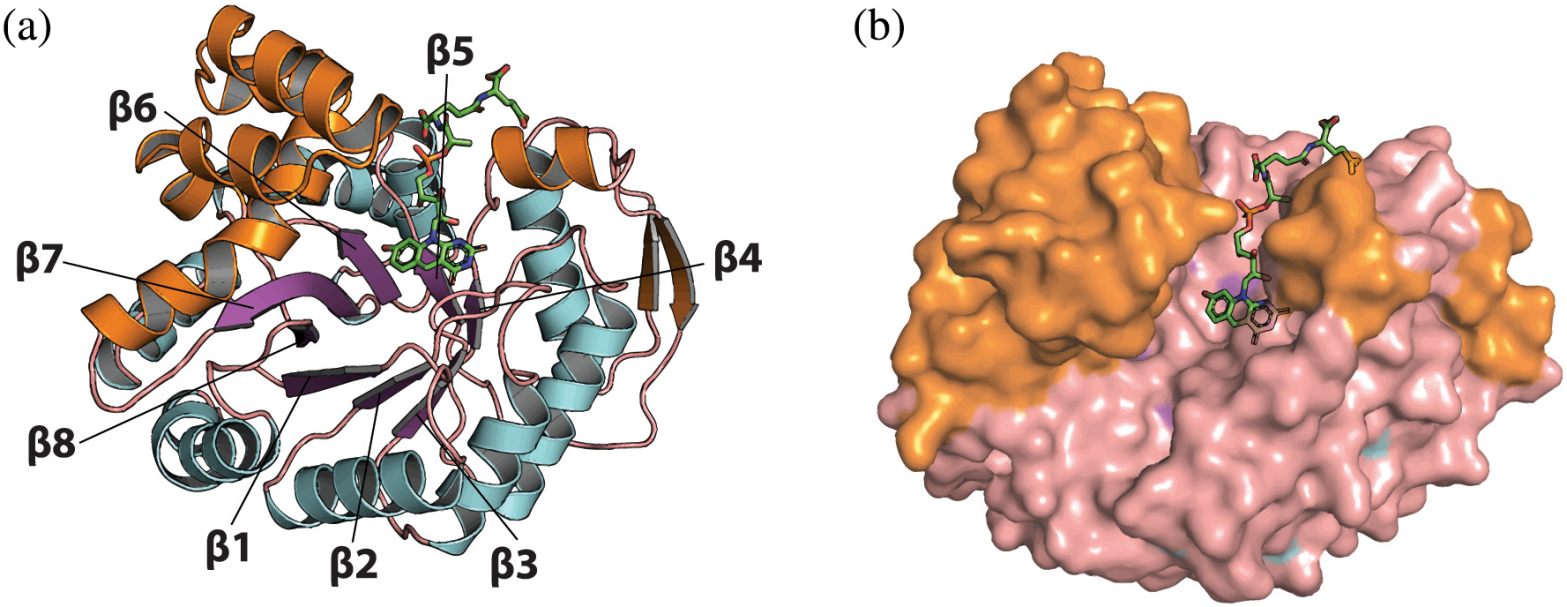
Structure of jMer in complex with F_420_. (a) Cartoon model. The β‐strands of the (βα)_8_ core unit are labeled and colored purple, while the α‐helices of the core unit are colored light blue and the loops are colored salmon. The inserted segments, which form a helical subdomain, are colored in orange. F_420_ is shown with the carbon in green. (b) Surface model with the stick model of F_420_. The color code is the same as in panel A.

Crystal structures for the apoenzyme of jMer and the binary jMer‐F_420_ complex were determined at a resolution of 1.8 and 1.9 Å, respectively (Table 2). The overall structure of jMer is almost identical to those of previously established methanogenic enzymes characterized by a (βα)_8_‐ or TIM‐barrel fold (Figure 2). The same fold was also found in the structures of MTHFR and Mfr (Gehl et al., 2023; Guenther et al., 1999).

**TABLE 2:** Structure determination statistics for the jMer apoenzyme and the binary complex of jMer and F_420_.

	jMer	jMer + F_420_
Resolution range (Å)	46.25–1.8 (1.86–1.8)	31.39–1.902 (1.97–1.902)
Space group	*P* 2_1_ 2_1_ 2_1_	*P* 4_1_ 2_1_ 2
Unit cell dimensions		
*a*, *b*, *c* (Å)	96.66, 96.28, 166.78	95.91, 95.91, 166.02
*α*, *β*, *γ* (°)	90, 90, 90	90, 90, 90
Unique reflections^a^	144,123 (14203)	60,825 (5895)
Completeness (%)^a^	99.84 (99.54)	98.83 (97.16)
Wilson *B*‐factor	33.65	47.64
Reflections used in refinement	144,109 (14202)	60,822 (5895)
Reflections used for *R*_free_	2002 (200)	1998 (194)
*R*_work_ (%)^b^	17.86 (27.11)	21.95 (32.62)
*R*_free_ (%)^b^	19.64 (29.09)	25.25 (33.64)
Protein residues	1324	657
RMSD bond lengths (Å)^c^	0.008	0.014
RMSD bond angles (°)^c^	1.07	1.36
Ramachandran favored (%)	97.02	96.01
Ramachandran allowed (%)	2.45	3.84
Ramachandran outliers (%)	0.54	0.15
Rotamer outliers (%)	0.19	1.16
Clash score	4.11	6.58
Average *B*‐factor	53.52	60.38
PDB code	8QPM	8QPL

^a^ Values relative to the highest resolution shell are written in parentheses.

^b^ *R*_free_ was calculated for 5% of the reflections that were not included in the refinement.

^c^ Root mean square deviation.

A few strand‐to‐helix loops crucially forms the active site architecture of TIM barrel enzymes (Farber & Petsko, 1990; Wierenga, 2001). The active site of jMer is located in a cleft between the α/β‐barrel domain and a helix‐bundle subdomain composed of five helices. In the experimentally determined jMer‐F_420_ structure, F_420_ is associated with the α/β‐barrel domain with the isoalloxazine ring positioned at the bottom of the cleft in contact with the C‐terminal loops of most strands of the (αβ)_8_ barrel (Figure 2a). The residual part of F_420_ pointing to the entrance of the cleft is sandwiched between the loops after β4 and β5 and reached the N‐terminal ends of the following helices (Figure 2b).

The cleft is sufficiently large to also accommodate methylene‐H_4_MPT with the pterin head adjacent to the isoalloxazine ring of F_420_. To study the hydride transfer of Mer, we tried to co‐crystallize jMer with F_420_/F_420_H_2_ and methylene‐/methyl‐H_4_MPT. However, the C_1_ carrier could not be found in the resulting electron density. Therefore, we built a model of the ternary complex of jMer for studying the catalytic mechanism.

### 2.3 Ternary complex model building

**FIGURE 3:**
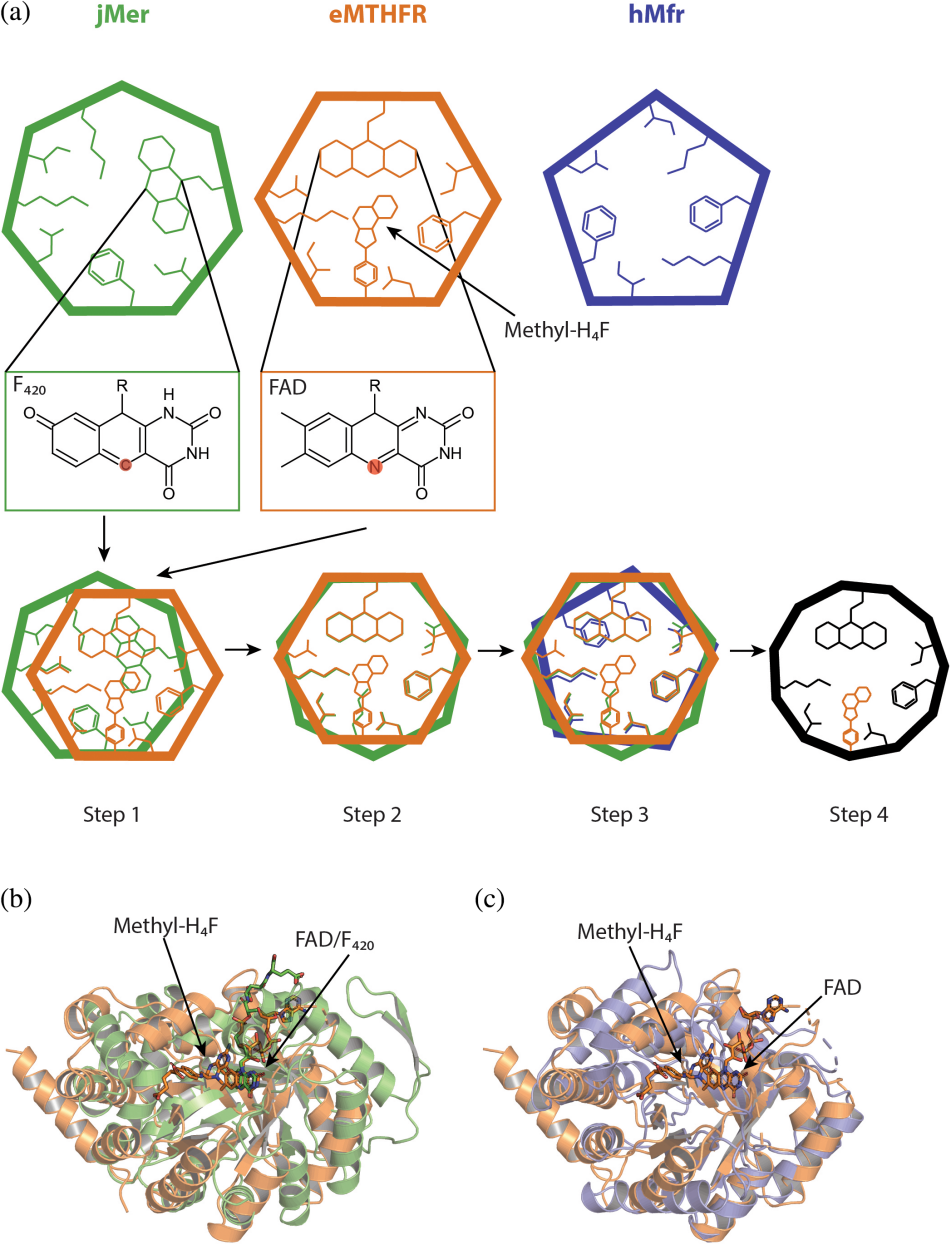
Alignment of three methylene‐tetrahydropterin reductases. (a) Methods of superposition. To align jMer on eMTHFR the hydride‐bearing atoms N5 of FAD and C5 of F_420_, highlighted in red, were laid on top of each other (step 1). Then, jMer was rotated on eMTHFR by fixing two hydride‐bearing atoms (step 2). hMfr was coordinate‐based aligned on eMTHFR in the normal manner (step 3). Finally, methyl‐H_4_F from eMTHFR was transferred into the superimposed jMer and hMfr. Identical amino‐acid residues were determined based on the performed structural alignment (step 4). The result of the alignment of jMer (green) onto eMTHFR (orange) (b), and Mfr (blue) onto eMTHFR (orange) (c). Methyl‐H_4_F and FAD of eMTHFR are shown by a stick model with carbon in orange. F_420_ of jMer is shown by a stick model with carbon in green.

**FIGURE 4:**
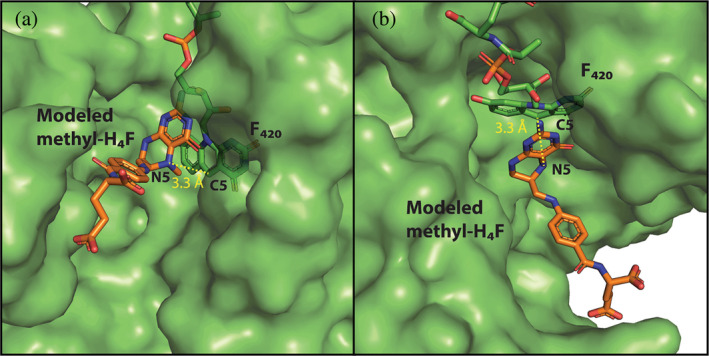
Ternary complex model of jMer based on the described alignment procedure. The polypeptide is drawn in a green surface representation, F_420_ (carbon in green) and methyl‐H_4_F (carbon in orange) as sticks (see Figure 3). The N5‐methyl group of methyl‐H_4_F is shown in red. The active‐site structure is shown from different angles (a and b). Although Mer uses methylene‐/methyl‐H_4_MPT as the native cofactors, methyl‐H_4_F was modeled based on the alignment strategy.

To compare the active site structure of eMTHFR, hMfr and jMer, the monomeric protein structures were superposed using a three‐dimensional alignment (Figure 3). A simple three‐dimensional alignment was not sufficiently accurate because of the large differences between the tertiary structures of jMer and the other two reductases (Figure S4). For that reason, the known highly related hydride transfer geometry between FADH_2_ and methylene‐H_4_F in eMTHFR, and F_420_H_2_ and methylene‐H_4_MPT in jMer was applied as additional information for alignment. Accordingly, the hydride‐bearing atoms of FAD (N5) in the eMTHFR structure and of F_420_ (C5) in the jMer structure were, at first, superposed (Figure 3, step 1) and then the rest of the proteins was aligned without moving the hydride‐transferring atoms (Figure 3, step 2). The hMfr and eMTHFR structures could be superimposed by the normal overall procedure due to their higher structural similarities (Gehl et al., 2023) (Figure 3, step 3). The root mean square deviation (RMSD) values were 4.7 Å between eMTHFR and hMfr (over 240 amino acids), 4.6 Å between hMfr and jMer (over 232 amino acids), and 5.3 Å between jMer and eMTHFR (over 200 amino acids). After superposition, identical or similar amino acids at the same position in space were identified and the methyl‐H_4_F of eMTHFR was transferred to hMfr and jMer (Figure 3, step 4). In jMer, the distance between C11 of the modeled methyl‐H_4_F and C5 of F_420_ is 3.3 Å (Figure 4). In the modeled jMer‐F_420_‐methyl‐H_4_F complex, methyl‐H_4_F do not substantially interfere with amino acid residues and its tail has no contact with the protein. In the ternary complex model of jMer shown in this work, methyl‐H_4_F was present as substrate rather than methyl‐H_4_MPT based on the alignment strategy. hMfr and eMTHFR were superimposed and methyl‐H_4_F and FAD was modeled to hMfr.

**FIGURE 5:**
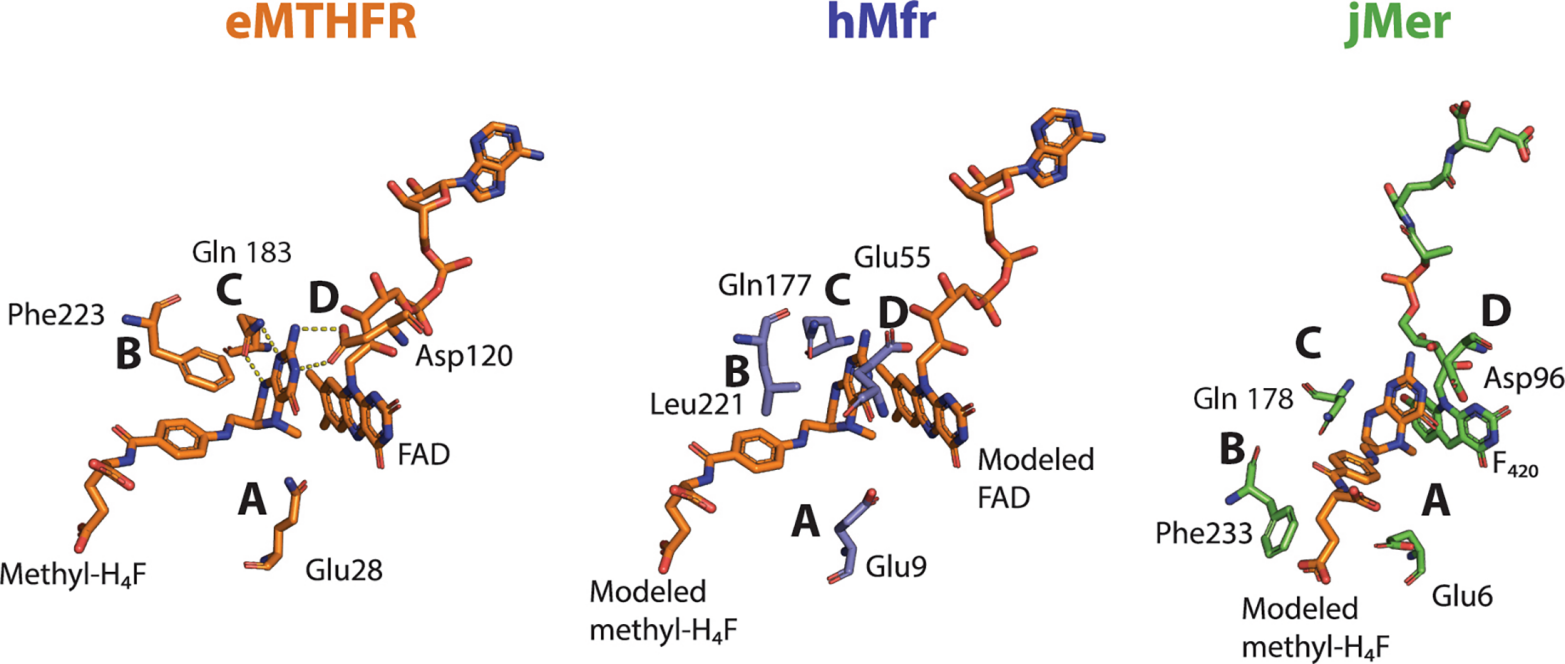
Comparison of the tetrahydropterin binding sites of the crystal structure of the ternary eMTHFR complex and the ternary complex models of hMfr and jMer. Carbons of residues of eMTHFR, hMfr, jMer are colored in orange, blue and green. Equivalent positions are indicated by letters. Non‐native coenzymes were modeled in the structure of hMfr and jMer based on the alignment strategy. This is the case for hMfr (using NADH, here modeled with FAD) and jMer (producing methyl‐H_4_MPT as a natural product, here modeled with methyl‐H_4_F).

Despite the structural difference between H_4_F and H_4_MPT in the model of jMer, we discuss the possible interactions of the protein and methyl‐H_4_MPT from the pterin head group to the phenyl ring moiety, which is structurally similar in H_4_F and H_4_MPT. A hydrophobic pocket consisting of the conserved residues Phe233, Val8 and Val230 might be responsible for binding of the phenyl ring of H_4_MPT in jMer. Phe233 is rather distant from the phenyl ring of the tail of H_4_MPT (Figure S5). However, a conformational change induced by binding of methylene‐H_4_MPT may be possible. Gln178 is located close to the bottom of the cleft at the C‐terminal end of β6, which is presumably hydrogen‐bonded with the F_420_ isoalloxazine and the pterin ring. Asp96 protruding from the C‐terminal end of β4 is also in contact with the pyrimidine ring of the C_1_ carrier. Glu6 at the C‐terminal end of β1 points towards the deaza‐isoalloxazine and pterin rings. The mentioned amino acids involved in binding of the pterin part of the C_1_ carrier have spatial counterparts in MTHFR and hMfr. The four sites for Glu6, Phe233, Gln178 and Asp96 are referred to as positions A‐D (in Figure 5). Positions A and D are occupied by acidic residues, position C by amino acids with carboxamide groups, and position B by large hydrophobic residues.

### 2.4 Mutational analysis of the C_1_ carrier binding site

Similar amino acid residues at the position A to D of the three methylene‐tetrahydropterin reductases indicate common active site characteristics, despite their insignificant amino acid sequence identity. Structure‐independent sequence comparison indicated that jMer shares 16% and 12% sequence identity with eMTHFR and Mfr, respectively, and the identity between hMfr and eMTHFR is 17%. A structure‐based alignment was also performed and presented in Figure S6. To explore whether the amino‐acid residues at position A, B, C and D are involved in binding of the C_1_ carrier and in catalysis, systematic mutational analyses were performed and the kinetic data were compared with those for the eMTHFR variants reported in the literature (Tables 3 and 4). The common reaction between MTHFR and the other two reductases (Mer and Mfr) is the oxidative half reaction of MTHFR, which does not involve the primary hydride carrier NADH. Therefore, we do not discuss the effect of the mutations of eMTHFR on the reactivity with NADH despite their influence on *k*_cat_ and *K*_m_ of NADH (see Tables 3 and 4 and Figure S7).

**TABLE 3:** Kinetic constants of eMTHFR, hMfr and jMer.

Enzyme	Substrate	App. *K*_m_ [μM]	App. *k*_cat_ [min^−1^]	*k*_cat_/*K*_m_ [min^−1^ μM^−1^]	Reference
eMTHFR	Methylene‐H_4_F	0.4 ± 0.1	130 ± 12	330	(Trimmer et al., 2005)
	NADH	3.5 ± 0.6		37	
	Methylene‐H_4_F	0.5 ± 0.1	620 ± 60^a^	1200	(Lee et al., 2009)
	NADH	20 ± 4		30	
hMfr	Methylene‐H_4_F	160 ± 62	600 ± 68	3.9 ± 0.8	(Gehl et al., 2023)
	NADH	16.0 ± 3.1	550 ± 19	35 ± 4.4	
jMer	Methylene‐H_4_MPT	58 ± 29.7	18,000 ± 4000	350 ± 90	This work
	F_420_	4.6 ± 0.1	7000 ± 30	1500 ± 20	

*Note*: The values for eMTHFR were obtained from the literature.

Abbreviation: nd, not determined.

**TABLE 4:** Kinetic constants for the variants of eMTHFR, hMfr and jMer.

Position	Enzyme	Mutation [reference]	Substrate	App. *K*_m_ [μM]	App. *k*_cat_ [min^−1^]	*k*_cat_/*K*_m_ [min^−1^ μM^−1^]
A	eMTHFR	Glu28Gln (Trimmer et al., 2001)	Methylene‐H_4_F	nd	<0.012 (0.002%)^a^	nd
			NADH	nd		nd
	hMfr	Glu9Gln (Gehl et al., 2023)	Methylene‐H_4_F	250 ± 60 (156%)	1.3 ± 0.1 (0.2%)	0.005 ± 0.001 (0.1%)
			NADH	43 ± 12 (268%)	1.1 ± 0.1 (0.2%)	0.028 ± 0.004 (0.1%)
	jMer	Glu6Gln	Methylene‐H_4_MPT	34 ± 10 (58%)	71 ± 8 (0.4%)	2.2 ± 0.3 (0.6%)
			F_420_	4.3 ± 1 (93%)	36 ± 2 (0.5%)	8.9 ± 1.8 (0.6%)
B	eMTHFR	Phe223Ala (Lee et al., 2009)	Methylene‐H_4_F	93 ± 16 (18,600%)^a^	170 ± 30 (28%)^a^	nd
			NADH	140 ± 7 (700%)^a^		nd
		Phe223Leu (Lee et al., 2009)	Methylene‐H_4_F	8 ± 2 (1600%)^a^	840 ± 120 (135%)^a^	nd
			NADH	240 ± 40 (1180%)^a^		nd
	hMfr	Leu221Phe	Methylene‐H_4_F	360 ± 30 (219%)	180 ± 6 (31%)	0.51 ± 0.02 (13%)
			NADH	3.4 ± 0.8 (21%)	89 ± 1 (16%)	26.9 ± 5.1 (78%)
		Leu221Ala	Methylene‐H_4_F	700 ± 120 (433%)	130 ± 10 (21%)	0.18 ± 0.01 (5%)
			NADH	5.2 ± 0.5 (33%)	38.0 ± 0.3 (7%)	7.3 ± 0.6 (21%)
	jMer	Phe233Ala	Methylene‐H_4_MPT	100 ± 50 (180%)	2200 ± 540 (12%)	23.1 ± 4.2 (7%)
			F_420_	1.8 ± 2.1 (39%)	470 ± 60 (7%)	377 ± 814 (25%)
		Phe233Leu	Methylene‐H_4_MPT	150 ± 100 (267%)	13,000 ± 4700 (73%)	97.9 ± 23.4 (28%)
			F_420_	3.1 ± 2.6 (67%)	1700 ± 240 (24%)	1400 ± 1300 (90%)
C	eMTHFR	Gln183Glu (Zuo et al., 2018)	Methylene‐H_4_F	110 ± 36 (27,000%)^a^	160 ± 30 (123%)^a^	nd
			NADH	6.6 ± 0.7 (189%)^a^		nd
		Gln183Ala (Zuo et al., 2018)	Methylene‐H_4_F	100 ± 10 (26,000%)^a^	17 ± 0.6 (13%)^a^	nd
			NADH	<10 (<286%)^a^		nd
	hMfr	Gln177Glu	Methylene‐H_4_F	380 ± 30 (236%)	100 ± 3 (17%)	0.26 ± 0.01 (7%)
			NADH	120 ± 40 (769%)	110 ± 15 (20%)	0.9 ± 0.1 (3%)
		Gln177Ala	Methylene‐H_4_F	570 ± 60 (347%)	56 ± 3 (9%)	0.1 ± 0.0 (3%)
			NADH	28 ± 7 (176%)	24 ± 1 (4%)	0.9 ± 0.1 (3%)
	jMer	Gln178Glu	Methylene‐H_4_MPT	20 ± 5 (34%)	430 ± 30 (2%)	22.3 ± 3.1 (6%)
			F_420_	27 ± 7 (593%)	510 ± 50 (7%)	18.9 ± 1.9 (1%)
		Gln178Ala	Methylene‐H_4_MPT	23 ± 10 (40%)	3.3 ± 0.5 (0.02%)	0.2 ± 0.0 (0.1%)
			F_420_	61 ± 67 (1326%)	6.1 ± 3.7 (0.1%)	0.03 ± 0.17 (0.002%)
D	eMTHFR	Asp120Asn (Trimmer et al., 2005)	Methylene‐H_4_F	17 ± 3 (4250%)^a^	0.44 ± 0.04 (0.3%)^a^	0.026 (0.01%)
			NADH	<3.5 (<100%)^a^		
	hMfr	Glu55Gln	Methylene‐H_4_F	180 ± 60 (108%)	180 ± 20 (30%)	1.1 ± 0.2 (28%)
			NADH	5.6 ± 1.1 (35%)	134 ± 2 (25%)	25 ± 4 (71%)
	jMer	Asp96Asn	Methylene‐H_4_MPT	140 ± 30 (246%)	700 ± 50 (4%)	4.9 ± 0.4 (1%)
			F_420_	1.6 ± 2.8 (35%)	85 ± 9 (1%)	5 ± 48 (0.3%)

*Note*: References for the eMTHFR variants and the published hMfr variants are listed below the variant name in parenthesis. The ratio of the *K*_m_, *k*_cat_, and *k*_cat_/*K*_m_ values of the variant compared to wild type is written below the absolute values in parenthesis.

Abbreviation: nd, not determined.

^a^ The kinetic parameters were compared with those of the wild‐type described in the references specified in the row of this table.

Position A contains a glutamate residue in all three reductases. For eMTHFR, the *k*_cat_ value of the Glu28Gln variant is reduced from 132 to 0.012 min^−1^ (0.01% residual activity). It has been proposed that Glu28 is the key catalytic residue for protonation of methylene‐H_4_F to activate the C_1_ unit for the reaction (Trimmer et al., 2001). In hMfr and jMer, apparent *k*_cat_ of the equivalent Glu9Gln and Glu6Gln variants decreased from 594 to 1.3 min^−1^ (0.2%) (Gehl et al., 2023), and from 18,200 to 71 min^−1^ (0.4%), respectively. Neither variant showed a significant increase in the apparent *K*_m_ for their respective C_1_ carrier, suggesting that glutamate and glutamine bind in a related manner. X‐ray structure analysis of the jMer_E6Q and hMfr_E9Q variants (Table S1) were performed and the structures were compared with the wild‐type enzyme (Figure S8), which confirmed unchanged active site architectures compared with the wild‐type enzymes.

Position B in eMTHFR is occupied by Phe223 whose side chain forms π–π interactions with the phenyl ring of methyl‐H_4_F. Mutation of Phe223 to alanine or leucine in eMTHFR is associated with a dramatic increase in the *K*_m_ values for methylene‐H_4_F and NADH without substantially changing the *k*_cat_ values (Lee et al., 2009). jMer carries a conserved Phe233 at position B but the exchange to alanine or leucine changed the apparent *K*_m_ values only slightly. The small increase of the apparent *K*_m_ values in the Phe233 variants in jMer indicate its participation in binding of the C_1_ carrier. However, the formation of strong π–π interactions as described for eMTHFR are unlikely for jMer due to the large distance between the phenyl ring moieties (see Figure 5). In hMfr, position B is occupied by the strictly conserved Leu221. Exchange of Leu221 to phenylalanine or alanine increased the *K*_m_ values of methylene‐H_4_F 2–4‐fold, which is again small compared with those of the eMTHFR variants. This result supports the hypothesis that Leu221 is involved in the binding of methylene‐H_4_F but with a different strength compared with that of eMTHFR.

Position C in the ternary complex structure of eMTHFR is occupied by Gln183, whose side chain carboxamide forms a bidentate hydrogen bond with NH8 and N1 (Pejchal et al., 2005). eMTHFR_Gln183Glu and Gln183Ala mutations are associated with a large increase in *K*_m_ values for methylene‐H_4_F (>250 fold). In contrast, the Gln183Glu mutation caused no change of the *k*_cat_ value (Zuo et al., 2018). The exchange of Gln177 in hMfr to glutamate or alanine moderately increased the apparent *K*_m_ values for methylene‐H_4_F (2–3‐fold) compared with that of eMTHFR. Notably, the apparent *K*_m_ value for NADH increased in hMfr_Gln177Glu (8‐fold), suggesting that the acidic side chain also influences the binding of NADH. In contrast, the replacement of Gln178 to glutamate and alanine in jMer slightly decreases the apparent *K*_m_ values for methylene‐H_4_MPT (30%–40%). Although H_4_F and H_4_MPT share NH8 and N1 of the pterin ring the interactions between the residue in position C and the pterin ring significantly differ between the three enzymes. In the jMer_Gln178 variants, the apparent *K*_m_ values for F_420_ substantially increased (6–13 fold). Since the backbone of Gln178 is part of the F_420_ binding site in jMer, the Gln178 variation may primarily affect the binding of F_420_ and not the binding of methylene‐H_4_MPT.

At position D, the carboxy group of Asp120 forms a bidentate hydrogen bond to N3 and the exo‐NH2 group of methyl‐H_4_F in the ternary complex of eMTHFR (Pejchal et al., 2005). The mutation of Asp120 to asparagine resulted in a large increase (>40 fold) of the *K*_m_ values for the C_1_ carrier in eMTHFR and a large decrease to 0.3% of the *k*_cat_ value (Trimmer et al., 2005). In the structure of hMfr, a glutamate rather than aspartate residue is located at the equivalent position. The hMfr_Glu55Gln variant did not show a significant increase in the apparent *K*_m_ value for methylene‐H_4_F but a decrease in the apparent *k*_cat_ value. In the case of jMer_Asp96Asn, the apparent *K*_m_ value was increased 2.5‐fold. These results suggest that the function of aspartate at position D in binding of the C_1_ carrier deviates in eMTHFR compared with the other two reductases. For hMfr, Glu55 is not strictly conserved and alanine and valine are found at this position in addition to glutamate and aspartate, which also supports this conclusion.

**FIGURE 6:**
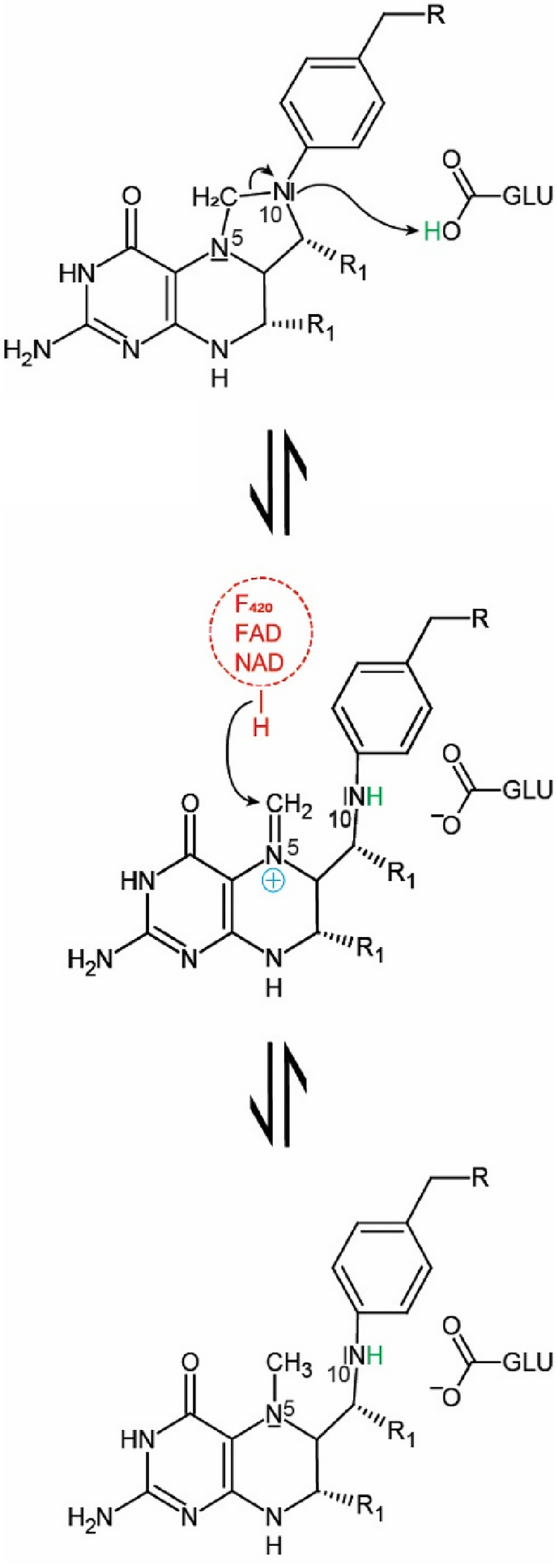
Postulated common catalytic mechanism for the three methylene‐tetrahydropterin reductases. In the first step, N10 of the pterin part of the C_1_ carrier is protonated leading to the formation of an minimum cation. The positive charge of the minimum cation is delocalized over the pterin ring system, presumably stabilizing the intermediate state. The positively charged minimum cation is an excellent hydride acceptor. After hydride transfer, the reaction product is obtained. R1 is a hydrogen or a methyl group in H_4_F and H_4_MPT, respectively.

Trimmer et al. proposed that methylene‐H_4_F is activated by protonation of N10 to form an N5 minimum cation (Trimmer et al., 2001). Based on the mutational analysis at position A, it was possible to extend the proposed model of the catalytic mechanism of MTHFR (Trimmer et al., 2001) not only to Mfr (Gehl et al., 2023) but also to Mer. The mechanism is initiated by the protonation of the C_1_ carrier to activate this molecule, followed by a hydride transfer from the hydride donor to the activated C_1_ unit (Figure 6). As the methylene group in methylene‐H_4_F and methylene‐H_4_MPT is chemically a rather unreactive aminal, it is unlikely that a hydride can be directly transferred. Therefore, the formation of a positively charged 5‐iminium cation is crucial for catalysis. The positive charge can be delocalized via the pterin ring system, which stabilizes this intermediate state. In contrast to the uncharged methylene group, the positively charged 5‐iminium cation is a good hydride acceptor for a hydride of either FADH_2_ in MTHFR, F_420_H_2_ in Mer or NADH in Mfr. On the other hand, even though some identical or similar residues were observed in the three methylene‐tetrahydropterin reductases at the predicted binding site of the C_1_ carriers at positions B–D, the kinetic effects of mutations argue against common features for the binding of the C_1_ carriers. An inaccurate modeling of methylene‐tetrahydropterin due to substrate‐induced conformational changes of contacting polypeptide segments and a consecutive bias in the interpretation of different kinetic behaviors cannot be fully excluded. On the other hand, the substantial changes of the kinetic values argue for a participation of the four residues, in particular, of the glutamate of position A.

### 2.5 Mutational analysis of the non‐prolyl *cis*‐peptide bond

**FIGURE 7:**
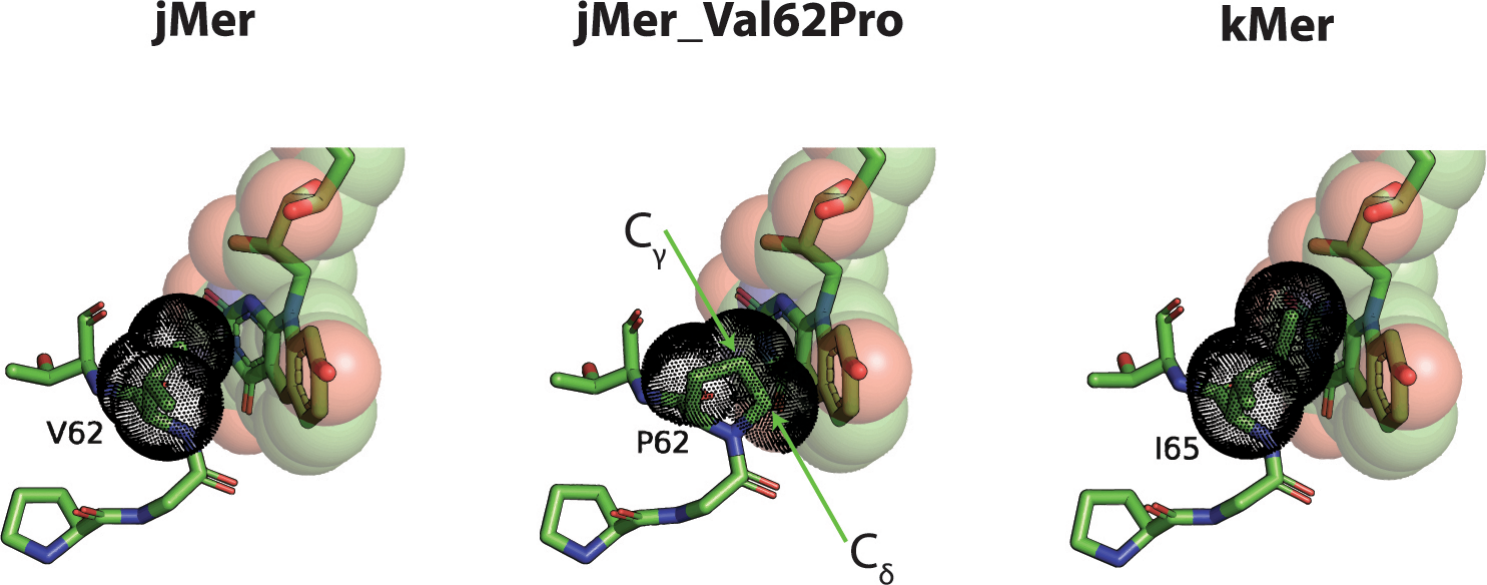
The jMer‐F_420_ complex, the AlphaFold model of jMer_Val62Pro, which contains prolyl *cis*‐peptide (PCP) and modeled F_420_, and the crystal structure of Mer from *M. kandleri* (kMer). F_420_ and the amino acids are shown as stick models with green carbons. F_420_ was also presented by drawing the corresponding atoms as transparent spheres with van der Waals radii and the *cis*‐peptide bond regions as black dotted spheres. F_420_ in kMer was modeled by alignment of the whole protein with the jMer structure in complex with F_420_. The non‐prolyl *cis*‐peptide bond of kMer is placed between Gly64 and Ile65. The side chain of the isoleucine in kMer does not overlap with modeled F_420_, whereas the side chain of proline in jMer_Val62Pro collides with F_420_.

Non‐prolyl *cis*‐peptide (NPCP) bonds are rare in proteins, but they play an important role when they do occur (Jabs et al., 1999; Pal & Chakrabarti, 1999). The NPCP bond in jMer is located in the loop after β3, below the central pyridine ring of F_420_, between Gly61 and Val62 (Figure 7). This NPCP bond is conserved at the equivalent position in all known Mer structures and even in other, for example, in the F_420_‐dependent glucose‐6‐phosphate dehydrogenase (Bashiri et al., 2008) and the F_420_‐dependent alcohol dehydrogenase (Aufhammer et al., 2004), but not in all enzymes of the bacterial luciferase superfamily. It has been proposed that the NPCP bond acts as a backstop for the placement of F_420_ in the active site (Aufhammer et al., 2005). In the present study, the role of this NPCP bond was tested by exchanging Val62 to Pro62, presumably resulting in conversion from the NPCP bond to a prolyl‐*cis* peptide (PCP) bond. The turnover number of the purified jMer_Val62Pro variant enzyme was 3.2 min^−1^ that is much lower than wild‐type jMer (5700 min^−1^) under standard assay conditions.

The AlphaFold model of jMer_Val62Pro supported the predicted PCP bond by a very high pLDDT of over 98 in the region (P60–T63) (Figure 7). Alignment of the jMer wild type binary complex structure and the jMer_Val62Pro model showed that the backbone of the corresponding loop and the C_β_ atoms of Val62 and Pro62 remain in the same position after mutation (Figure 7). However, C_γ_ and C_δ_ of Pro62 occupy the space of F_420_ in the conformation of the wild type enzyme. Although the backstop function of the *cis*‐peptide conformation is maintained, the ability to bind F_420_ is thereby substantially reduced. Spatial consideration indicated that hydrophobic residues of moderate size can replace Val62. Indeed, valine is not strictly conserved at this position and isoleucine is found in other Mer enzymes, e.g. in Mer of *M. kandleri* (Figure 7) (Shima et al., 2000). Larger side chains cannot be placed at this position because they would interfere with the loop after α4, which is involved in the binding of the first two hydroxy groups of the F_420_ tail region. Small side chains, such as in alanine, do not reach F_420_ and cannot exert pressure to adjust the conformation of F_420_. This analysis comprehensibly demonstrates that proteins can realize an essential biological function by introducing an energetically unfavorable NPCP bond, which cannot be achieved by a PCP bond.

### 2.6 Phylogenetic analyses of methylene‐tetrahydropterin reductases

**FIGURE 8:**
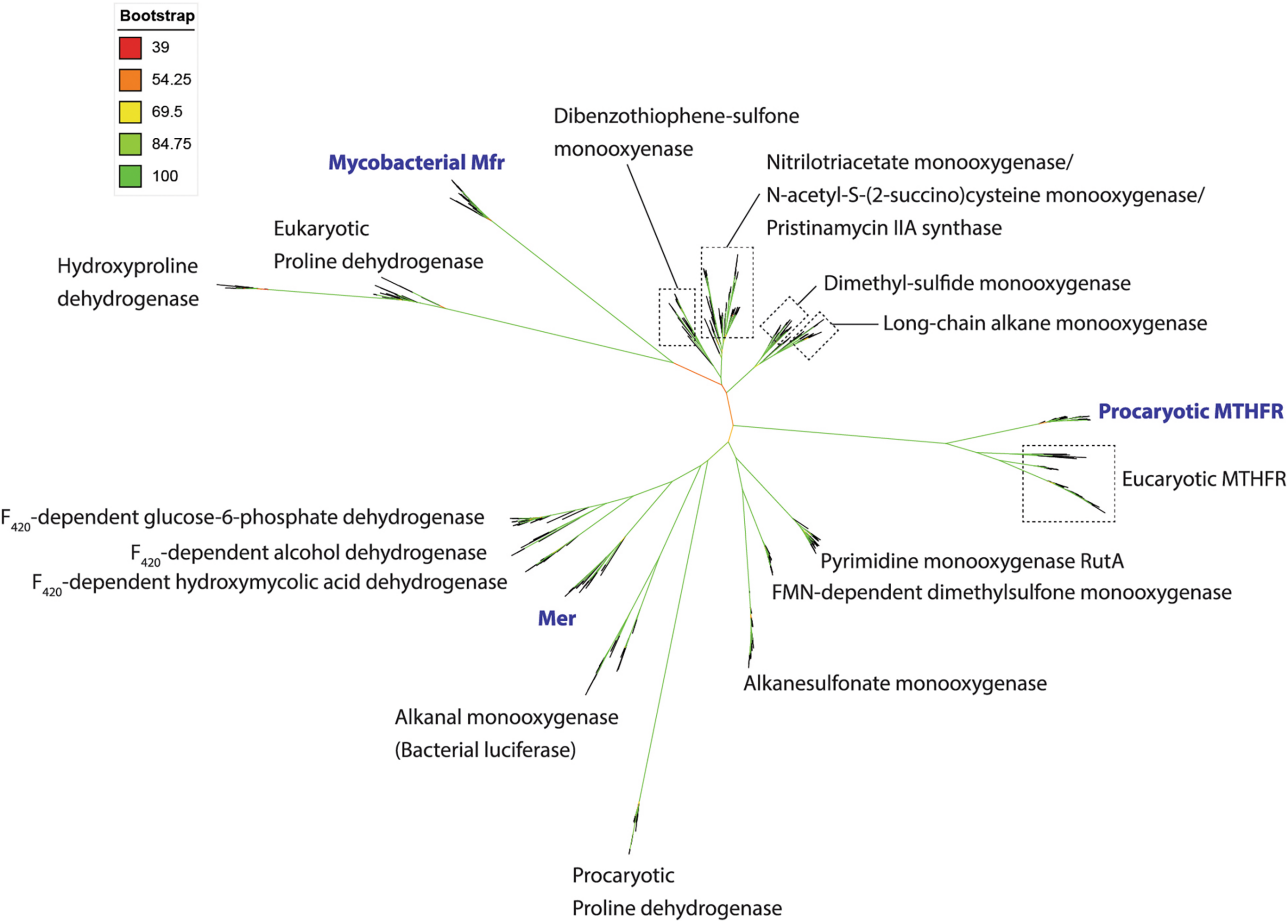
Unrooted phylogenetic tree of bacterial luciferase superfamily members, flavin‐linked oxidoreductase superfamily members and Mfr members. The bootstrap values are indicated by a color scheme. The central part of the tree does not allow final conclusions about the basal relationship between the tree superfamilies.

To obtain further insights into the phylogenetic relationship of the three methylene‐tetrahydropterin reductases, a phylogenetic tree was constructed (Figure 8) using sequences from the bacterial luciferase family including Mer (Mer superfamily; Table S2), the FAD‐dependent methylene‐H_4_F reductase superfamily (MTHFR superfamily; Table S3), and Mfr for BLAST searches.

The Mer cluster, placed next to bacterial luciferases and F_420_‐dependent dehydrogenases (Mascotti et al., 2021), the MTHFR cluster, consisting of prokaryotic and eukaryotic FAD‐dependent representatives, and the mycobacterial Mfr cluster are isolated in the tree with no reliable connection between each other and to any other clusters. Moreover, the bottom of the bacterial luciferase family in the phylogenetic tree is not occupied with a Mer‐like methylene‐H_4_MPT reductase indicating that the ancestor of Mer does not reduce methylene‐H_4_MPT. A different function is also plausible for the last universal MTHFR ancestor, as on one hand its hypothetical flavin dependency was developed at the branch bottom suggested by the close clustering of MTHFRs widespread in all domains of life, and on the other by the parallel worthlessness of the prosthetic group for MTHFR for catalysis, because simpler Mfr without flavin can execute the same reaction. Therefore, FAD in MTHFR may only serve as an evolutionary relict. Obscured by the lack of sequence and biochemical data, the same might be true for Mfr. In addition, a global phylogenetic analysis of the whole metabolism of C_1_‐unit reduction results in an unrelated biosynthesis of H_4_MPT and H_4_F (de Crecy‐Lagard et al., 2012; Maden, 2000; Sousa & Martin, 2014) and in non‐homologous enzymes catalyzing the reduction series for C_1_ units bound to H_4_F and H_4_MPT (Martin & Russell, 2007; Sousa & Martin, 2014). These factors would altogether support an independent development of the three enzymes, at least, of Mer with respect to the others. It has to be kept in mind that the separation of the MTHFR and Mfr clusters in the tree may result from missing links due to the absence of data towards the bacterial luciferase superfamily, as the sequences of Mfr are very limited and no Mfr candidates have been investigated outside the order *Mycobacteriales*.

### 2.7 Divergent versus convergent evolution

The presented results indicated common and distinct features in the three methylene‐tetrahydropterin reductases for catalyzing the related hydride transfer reactions. This finding raises the question whether these three methylene‐tetrahydropterin reductases evolved from one common ancestor or converge from a different origin. From the mechanistic point of view both Mer and Mfr have in common a one‐step hydride transfer process suggesting a closer phylogenetic relationship. On the other hand, structural alignment clearly showed a stronger connection between MTHFR and Mfr.

In the literature, a divergent development of TIM barrels is tendentially favored as remote sequence relationships are detectable among most distinct superfamilies (Nagano et al., 2002). For the TIM barrel proteins Mer, Mfr and MTHFR the related bulky substrates arranged to each other in an analogous manner and the shared hydride transfer reaction also point in this direction. Furthermore, incomplete sequence data, similar kinetic effects of equivalent mutations in hMfr and eMTHFR rather than in jMer and hMfr or in jMer and eMTHFR and several structural features support the possibility that, at least, Mfr was developed from MTHFR by drastic divergence as found in the evolution of (βα)_8_‐barrels (Gerlt & Babbitt, 1998; Romero‐Romero et al., 2021; Sterner & Hocker, 2005). In this case, the flavin of MTHFR would be replaced by NADH in Mfr of mycobacteria later on.

On the other hand, various phylogenetic, mutational/kinetic and structural data rather argue for an independent origin due to the unrelated sequences and the largely different structures of segments directly involved in forming the substrate binding and active sites. This finding is striking, as the similar substrates and the equivalent chemical reactions with the same rate‐limiting transition state should not require such drastic structural changes between the three reductases. An independent origin is also taken into accounts, because TIM barrel folds are presumably manifold formed during evolution from smaller (αβ)_2_, and (αβ)_4_ building blocks by gene duplication and domain fusion (Lang et al., 2000). The correlating and non‐correlated kinetic data of the site‐specific variants of the shared glutamate as a key catalytic residue at position A and of the residues at positions B‐D, respectively, are compatible with a convergent and divergent evolution. Future biochemical characterizations of ancestral flavin‐dependent MTHFR, acetogenic FMN‐dependent MTHFRs and, in particular, of ancestral Mfr functional homologs may clarify the evolutionary relationship between Mer, Mfr, and MTHFR.

## 3 MATERIALS AND METHODS

### 3.1 Purification of H_4_MPT and F_420_

Approximately 130 g of *M. marburgensis* cells cultured under the standard nickel‐sufficient conditions were used to purify H_4_MPT as previously described (Shima et al., 2011). For the purification of F_420_, three cell pellets resulting from the H_4_MPT purification were combined and diluted 1:4 in anaerobic water. The suspension was sonicated using a SONOPLUS GM200 (Bandelin) with a VS‐70‐T tip attached at 80% power of 100 W for the whole period of 15 min. The suspension was mixed 1:1 with 100% acetone (−20°C) and stirred in an ice bath for 30 min. The mixture was centrifuged on at 13000 × *g* and 4°C for 20 min. The supernatant was collected and the extraction was repeated twice by adding 50% (v/v) aqueous acetone cooled to −20°C in a 1:1 ratio regarding the weight of cell debris. The combined supernatants were evaporated at 4°C until the volume has decreased by at least half. The evaporated solution was centrifuged as described above and applied to a QAE Sephadex A25 column (GE Healthcare) equilibrated with 500 mL of 50 mM Tris/HCl pH 7.5. The column was washed with 500 mL 50 mM Tris/HCl pH 7.5 and then with 500 mL 300 mM formic acid in water. Elution was performed with a single step gradient of 500 mL 50 mM HCl in water. The F_420_ containing fractions were combined and concentrated by evaporation. The concentrate was desalted using a Sephadex G‐10 column (Cytiva Life Sciences) equilibrated with water. The F_420_ solution was stored in aliquots at −20°C.

### 3.2 Mutagenesis and heterologous overproduction of jMer

The vector pT7‐7_jMer containing the jMer encoding gene *MJ1534* was used for targeted mutagenesis. The degenerated primers for mutagenesis were designed using NEBaseChanger. A PCR was performed using 10 ng pT7‐7_jMer as template, 0.5 μM degenerate primers (Table S4), 1× Q5 reaction buffer (New England Biolabs), 200 μM dNTPs (Thermo Fisher Scientific) and 0.02 U/μL Q5 High‐Fidelity DNA Polymerase (New England Biolabs) in a 50 μL reaction volume. Thermocycling conditions were selected according to the manufacturer's recommendations and the annealing temperature was used as recommended by the NEBaseChanger (Table S4). After PCR, the template DNA was digested with DPNI at 37°C for 1 h. The preparation was purified using the NucleoSpin Gel and PCR Clean‐up Kit (Macherey‐Nagel). The DNA preparation was used for transformation into chemically competent *E. coli* Top10 cells and the cell suspension was plated on agar plates containing 100 μg/mL carbenicillin. After colony formation, 5 mL of LB medium supplemented with 100 μg/mL carbenicillin was inoculated with one colony and grown overnight at 37°C. The plasmids were isolated using the NucleoSpin Plasmid Kit (Macherey‐Nagel) and sequenced by Eurofins using pT7‐7_Seq_F and pT7‐7_Seq_R as sequencing primers (Table S4). The correct plasmids were transformed into ArcticExpress (DE3) cells and plated on agar plates containing 100 μg/mL carbenicillin and 20 μg/mL gentamicin. One colony was inoculated into 5 mL of LB medium and incubated overnight at 37°C. A cryo‐culture was prepared by mixing 1 mL of 50% glycerol with 1 mL of the overnight culture and flash frozen. Cryo‐cultures were stored at −75°C.

A cryo‐culture of *E. coli* ArcticExpress(DE3) containing the desired jMer variant was used to inoculate 100 mL of LB medium supplemented with 100 μg/mL carbenicillin and 20 μg/mL gentamicin. The pre‐culture was incubated overnight at 37°C with shaking at 120 rpm. The main culture contained 2 liters of pre‐warmed TB medium supplemented with 100 μg/mL carbenicillin and 20 μg/mL gentamicin and was inoculated with 100 mL of the pre‐culture. The main culture was incubated at 37°C with stirring at 600 rpm until an optical density of 0.6–0.8 was reached. The *mer* gene expression was induced with 1 mM IPTG and the culture was transferred to 21°C. After 21 h of expression, the culture was harvested by centrifugation at 13,000 × *g* for 5 min at 4°C. Cells were snap frozen and stored at −20°C.

### 3.3 Purification of jMer

For crystallization, approximately 40 g of the *E. coli* cells were suspended in 160 mL of 50 mM Tris/HCl pH 7.5 with 2 mM DTT. The cell suspension was sonicated using a SONOPULS GM200 (Bandelin) with a 50% cycle and 160 W for 5 min per cycle and 5 min pause using a TZ76 tip. A total of 2 cycles were performed. The disrupted cells were fractionated by centrifugation at 30,000 × *g* for 30 min at 4°C. The supernatant was heated at 80°C for 20 min and precipitated proteins were separated by centrifugation at 13,000 × *g* for 20 min at 4°C. Ammonium sulfate was added to the supernatant to 60% saturation and the solution was stirred at 4°C for 20 min. Precipitated proteins were removed by centrifugation at 13,000 × *g* for 20 min at 4°C. The supernatant was applied to a Phenyl‐Sepharose HP column (15 mL column volume) equilibrated with 50 mM Tris/HCl pH 7.5 containing 2 mM DTT and 2 M ammonium sulfate (buffer A). Buffer B contained 50 mM Tris/HCl pH 7.5 with 2 mM DTT and 10% (v/v) glycerol. The column was washed with 20% buffer B. Elution was performed with a linear gradient from 20% to 100% buffer B in eight column volumes. jMer was eluted from 137 to 5 mS/cm conductivity. The corresponding fractions were collected and desalted on a HiPrep G‐25 column equilibrated with 50 mM Tris/HCl pH 7.5 containing 2 mM DTT. The desalted solution was applied to a Resource Q column (6 mL column volume) equilibrated with 50 mM Tris/HCl pH 7.5 with 2 mM DTT. jMer was eluted by a linear gradient of 0–250 mM NaCl over 15 column volumes. The jMer‐containing fractions were collected and applied to a HiPrep Sephacryl S‐200 HR equilibrated with 50 mM Tris/HCl pH 7.5 containing 150 mM NaCl and 2 mM DTT. The jMer‐containing fractions were either used directly for crystallization or, after the addition of 5% (v/v) glycerol, were snap frozen in liquid nitrogen and stored at −75°C.

A shorter protocol was developed for the characterization of jMer variants. Approximately 10 g of cells were suspended in 40 mL of 50 mM Tris/HCl pH 7.5 containing 2 mM DTT. The cell suspension was sonicated with a TZ73 tip attached to a SONOPULS GM200 (Bandelin) with a 50% cycle and 160 W for 5 min per cycle and 5 min pause. A total of 2 cycles were performed. The disrupted cells were fractionated by centrifugation at 30,000 × *g* for 30 min at 4°C. The supernatant was heated at 80°C for 20 min and precipitated proteins were separated by centrifugation at 13,000 × *g* for 20 min at 4°C. The supernatant was diluted 1:1 in 50 mM Tris/HCl pH 7.5 with 2 mM DTT and applied directly to a ResourceQ column (6 mL column volume) equilibrated with 50 mM Tris/HCl pH 7.5 with 2 mM DTT. jMer was eluted by a linear gradient of 0–250 mM NaCl over 15 column volumes. The jMer‐containing fractions were collected and used for the kinetic characterization.

### 3.4 Activity assay of jMer

Master mixes for the activity assays were prepared in an anaerobic chamber. Brown serum bottles were filled with 100 mM Tris/HCl pH 8.0 supplemented with 10 μM 2‐mercaptoethanol, the desired amount of purified F_420_ and H_4_MPT, and 3 mM sodium dithionite. The master mixes were incubated for 15 min at 55°C in a water bath, in which F_420_ was reduced to F_420_H_2_. The enzyme assay was performed in an anaerobic quartz cuvette (1 cm light path) with a final volume of 200 μL. After preheating at 55°C for 5 min, 15 mM formaldehyde (final concentration) was added, by which methylene‐H_4_MPT was generated from H_4_MPT and residual dithionite was quenched. Under the standard assay condition, the reaction mixture contained 20 μM methylene‐H_4_MPT and 20 μM F_420_H_2_ at 55°C. In the case of Michaelis–Menten kinetic analysis, one of the substrate concentration was fixed as the standard condition. The enzyme reaction was started by the addition of 10 μL of enzyme solution. The reaction was monitored by measuring the increase in absorbance at 401 nm. The catalytic activity was calculated from the extinction coefficient of F_420_ (*ε*_401_ = 25.9 mM^−1^ cm^−1^).

### 3.5 Crystallization and structure determination of jMer

All crystallization experiments were carried out in an anaerobic chamber with a 95%/5% (N_2_/H_2_) atmosphere using the sitting drop vapor diffusion method and 96‐well two‐drop MRC crystallization plates (Molecular Dimensions). The plates were incubated for 1 week in the chamber before use. The final protein concentration in each drop was 20 mg/mL. For the drops containing F_420_, a final F_420_ concentration of 2 mM was used. The crystal of the apoenzyme grew in a drop consisting of 35% (v/v) 2‐methyl‐2,4‐pentanediol and 100 mM sodium acetate pH 4.5 and could be frozen directly in liquid nitrogen. The best crystal of the binary complex grew in a drop consisting of 25% (v/v) polyethylene glycol monomethyl ether 550, 100 mM MES pH 6.5 and 10 mM zinc sulfate. Prior to freezing, the crystal was treated with a cryoprotectant solution consisting of the reservoir solution mixed with 20% polyethylene glycol monomethyl ether 550 and F_420_ to a final concentration of 2 mM. A large number of experiments were also carried out using F_420_ in combination with either methylene‐H_4_MPT or methyl‐H_4_MPT at concentrations ranging from 2 to 10 mM substrate concentration in the droplets.

The diffraction experiments were performed at 100 K on the SLS beamline X10SA (Villigen, Switzerland) equipped with a Dectris Eiger2 16M detector. The data set was processed with XDS and scaled with XSCALE (Kabsch, 2010). The phase problem was solved by the molecular replacement method using PHASER (McCoy et al., 2007) with the structure of Mer from *Methanopyrus kandleri* as a search model (Shima et al., 2000). The model was built and improved in COOT (Emsley et al., 2010) and refined using Phenix.refine (Liebschner et al., 2019) and Refmac (Murshudov et al., 2011). The final model was validated using the MolProbity (Williams et al., 2018) implementation of Phenix (Liebschner et al., 2019). Data collection, refinement statistics and PDB code for the deposited structure are listed in Tables 2 and S1.

### 3.6 Mutagenesis, expression, purification and activity assay of hMfr

hMfr variants were generated by GenScript. The expression, purification and activity assay procedure was performed as previously described (Gehl et al., 2023).

### 3.7 Kinetic data processing and figure generation

The graphs and analyses of the kinetic constants were carried out using Python 3.7 with Jupyter Notebook (version 6.1.4) (Kluyver et al., 2016) as the development environment and the following packages: os, pandas (McKinney & Data structures for statistical computing in python, 2010), seaborn (Waskom, 2021), matplotlib (Hunter, 2007), NumPy (Harris et al., 2020), SciPy (Virtanen et al., 2020). The code can be found at GitHub (https://github.com/ManuelGehl/Enzyme-kinetic-fitting). The chemical structures were created using ChemSketch and edited using Adobe Illustrator.

### 3.8 Phylogenetic tree construction and structure alignment

Seed sequences from the bacterial luciferase family (Table S2) and the FAD‐linked reductase superfamily (Table S3) together with the amino acid sequence of hMfr were used for separate BLAST searches against the clustered non‐redundant protein sequence database. The results were filtered to exclude any cluster with more than 90% sequence identity, and all clusters containing at least three members were selected. The query sequences were reinserted into the dataset and sequences marked as partial were removed, resulting in 500 sequences. A multiple sequence alignment was performed using MUSCLE (Edgar, 2004) and a maximum likelihood tree was constructed using IQTree (Minh et al., 2020). The best fitting evolutionary model was found to be WAG+F + I + G4 and the ultra‐fast bootstrap method (Hoang et al., 2018) was used to incorporate bootstrap values. The tree was visualized using iTOL (Letunic & Bork, 2021). Isolated branches were manually removed, resulting in 483 sequences in the tree. In addition, structure alignment was performed using PROMALS3D (Pei et al., 2008) as a tool and the crystal structures of hMfr, jMer and eMTHFR.

## AUTHOR CONTRIBUTIONS

**Manuel Gehl:** Conceptualization; software; data curation; formal analysis; investigation; methodology; validation; writing – original draft; writing – review and editing. **Ulrike Demmer:** Data curation; investigation. **Ulrich Ermler:** Conceptualization; funding acquisition; project administration; resources; data curation; formal analysis; investigation; methodology; software; validation; writing – review and editing. **Seigo Shima:** Conceptualization; funding acquisition; project administration; resources; supervision; writing – review and editing; writing – original draft.

## FUNDING INFORMATION

This work was supported by Max Planck Society (Ulrich Ermler and Seigo Shima) and by Deutsche Forschungsgemeinschaft, Priority Program, Iron–Sulfur for Life (SPP1927, SH87/1‐2) (Seigo Shima).

## CONFLICT OF INTEREST STATEMENT

The authors declare no competing financial interest.

## Supporting information


**TABLE S1.** Structure determination statistics for jMer_E6Q and Mfr_E9Q.
**TABLE S2.** Seed sequences for the superfamily of bacterial luciferases.
**TABLE S3.** Seed sequences for the superfamily of FAD‐linked reductases.
**TABLE S4.** List of primers used for mutagenesis of jMer and sequencing of the ORF of jMer in pT7‐7_jMer. The degenerated nucleotides are marked in red.
**FIGURE S2.** Size‐exclusion chromatography of jMer using a HiPrep Sephacryl S‐200 HR column. The peak of jMer is centered at around 50 mL elution volume and corresponds to a molecular mass of approximately 80 kDa.

**FIGURE S1:** Structures of H_4_MPT (top) and H_4_F (bottom). The pterin part is colored blue. The p‐aminobenzoate (PABA) ring of H_4_F and the aniline ring of H_4_MPT are colored red. The tail regions are colored black.

**FIGURE S3:** Dimeric structure of jMer. The homodimer is the physiological form of jMer and is formed by a two‐fold rotational axis at the center of the protein‐protein interface. The active site is located at the C‐terminal end of the parallel β‐strands, which are positioned on the opposite site of the two monomers.

**FIGURE S4:** Comparison of the tertiary structures of eMTHFR, jMer and hMfr. The β‐strands of the core unit are labeled and colored purple, while the α‐helices of the core unit and the loops are colored in light blue and salmon. The inserted helical segment is painted in orange. Methyl‐H_4_F and FAD are shown in orange and F_420_ is shown in green.

**FIGURE S5:** Hydrophobic pocked in jMer. The modeled H_4_F is colored yellow while the native F_420_ and the amino acids are shown in light blue.

**FIGURE S6:** Structure‐based alignment of hMfr, eMTHFR and jMer.

**FIGURE S7:** NADH binding site of eMTHFR.

**FIGURE S8:** (a) Comparison of the structures of jMer wild type (green) and jMer_E6Q (light blue). (b) Comparison of the structures of hMfr wild type (dark blue) and hMfr_E9Q (yellow). The glutamate residues are depicted as ball‐and‐stick model.

## Data Availability

Data available in article supplementary material.
