# Supplementary material for: Mutational and structural studies of (βα)8‐barrel fold methylene‐tetrahydropterin reductases utilizing a common catalytic mechanism
Source: Protein Sci. 2024 May 15;33(6):e5018. doi: 10.1002/pro.5018 (PMC11094777; doi:10.1002/pro.5018)
Supplement: Supplementary file 1 — TABLE S1. Structure determination statistics for jMer_E6Q and Mfr_E9Q. TABLE S2. Seed sequences for the superfamily of bacterial luciferases. TABLE S3. Seed sequences for the superfamily of FAD‐linked reductases. TABLE S4. List of primers used for mutagenesis of jMer and sequencing of the ORF of jMer in pT7‐7_jMer. The degenerated nucleotides are marked in red. FIGURE S2. Size‐exclusion chromatography of jMer using a HiPrep Sephacryl S‐200 HR column. The peak of jMer is centered at around 50 mL elution volume and corresponds to a molecular mass of approximately 80 kDa. [file PRO-33-e5018-s001.docx]

SUPPLEMENTARY INFORMATION

Table S1: Structure determination statistics for jMer_E6Q and Mfr_E9Q.

|  | jMer_E6Q | Mfr_E9Q |
| --- | --- | --- |
| **Resolution range (Å)** | 36.66 - 2.0 (2.072 - 2.0) | 45.8 - 1.75 (1.813 - 1.75) |
| **Space group** | *I* 4_1_ 2 2 | *P* 4_3_ |
| **Unit cell dimensions**  a, b, c (Å)  α, β, γ (°) | 98, 98, 200.78  90, 90, 90 | 45.8, 45.8, 254.6  90, 90, 90 |
| **Unique reflections ^a^** | 33180 (3259) | 51862 (5183) |
| **Completeness (%) ^a^** | 99.07 (98.72) | 98.91 (99.58) |
| **Wilson B-factor** | 46.17 | 25.15 |
| **Reflections used in refinement** | 33124 (3237) | 51850 (5183) |
| **Reflections used for R-free** | 1998 (196) | 1998 (196) |
| **R-work (%) ^b^** | 19.29 (39.87) | 19.97 (33.18) |
| **R-free (%) ^b^** | 23.44 (43.02) | 23.15 (39.49) |
| **Protein residues** | 331 | 586 |
| **RMSD bond lengths (Å) ^c^** | 0.007 | 0.006 |
| **RMSD bond angles (°) ^c^** | 0.90 | 0.84 |
| **Ramachandran favored (%)** | 96.96 | 98.79 |
| **Ramachandran allowed (%)** | 2.74 | 1.21 |
| **Ramachandran outliers (%)** | 0.30 | 0.00 |
| **Rotamer outliers (%)** | 0.77 | 0.00 |
| **Clash score** | 6.5 | 3.4 |
| **Average B-factor** | 64.5 | 33.8 |
| **PDB code** | 8QQ8 | 8QPJ |

^a^ Values relative to the highest resolution shell are within parentheses. ^b^ R_free_ was calculated for 5% of the reflections that were not included in the refinement. ^c^ rmsd, root mean square deviation.

Table S2: Seed sequences for the superfamily of bacterial luciferases.

| Family | Seed | Organism | Structural prototype | NPCP bond | Reducing agent |
| --- | --- | --- | --- | --- | --- |
| Bacterial luciferase/NFP (IPR002103) | Alkanal monooxygenase alpha chain (P07740) | *Vibrio harveyi* | PDB: 1LUC | A74-A75 | FMN |
|  | Alkanal monooxygenase beta chain (P07739) | *Vibrio harveyi* | PDB: 1LUC | No | None |
| Nitrilotriacetate monooxygenase component A/pristinamycin IIA synthase subunit A (IPR016215) | Long-chain alkane monooxygenase (A4IU28) | *Geobacillus thermodenitrificans* | PDB: 3B9N, 3B9O | No | FMN |
|  | Dimethyl-sulfide monooxygenase (E9JFX9) | *Hyphomicrobium sulfonivorans* | PDB: 6AK1 | No | FMN |
|  | N-acetyl-S-(2-succino)cysteine monooxygenase (P54950) | *Bacillus subtilis* | AlphaFold:AF-P54950-F1 | No | FMN? |
|  | Nitrilotriacetate monooxygenase component A (P54989) | *Aminobacter aminovorans* | PDB: 3sdo | No | FMN? |
|  | Pristinamycin IIA synthase subunit A (P54991) | *Streptomyces pristinaespiralis* | AlphaFold: AF-P54991-F1 | No | FMN? |
|  | Dibenzothiophene-sulfone monooxygenase (Q6WNP3) | *Rhodococcus erythropolis* | PDB: 5TLC | No | FMN |
| Alkanesulphonate monooxygenase, FMN-dependent (IPR019911) | Alkanesulfonate monooxygenase (A1A9L2) | *Escherichia coli* | PDB: 1M41 | No | FMN |
| Pyrimidine monooxygenase RutA (IPR019914) | Pyrimidine monooxygenase RutA (A1A9R7) | *Escherichia coli* | PDB: 5WAN | No | FMN |
| TAT-translocated F420-dependent dehydrogenase, FGD2 family (IPR031017) | F420-dependent hydroxymycolic acid dehydrogenase (P96809) | *Mycobacterium tuberculosis* | AlphaFold: AF-P96809-F1 | G111-V112 | F_420_ |
| FMNH(2)-dependent dimethylsulfone monooxygenase SfnG (IPR024014) | FMNH(2)-dependent dimethylsulfone monooxygenase (Q65YW9) | *Pseudomonas putida* | AlphaFold: AF-Q65YW9-F1 | No | FMN |
| 5,10-methylenetetrahydromethanopterin reductase (IPR019946) | 5,10-methylenetetrahydromethanopterin reductase (Q58929) | *Methanocaldococcus jannaschii* | This work | G61-V62 | F_420_ |
| F420-dependent glucose-6-phosphate dehydrogenase (IPR019944) | F420-dependent glucose-6-phosphate dehydrogenase (A0QLV0) | *Mycobacterium avium* | PDB: 3B4Y | S73-V74 | F_420_ |
| F420-dependent glucose-6-phosphate dehydrogenase-related (IPR019945) | Luciferase-like domain-containing protein (F420-dependent alcohol dehydrogenase Afd) (O93734) | *Methanoculleus thermophilus* | PDB: 1RHC | C72-I73 | F_420_ |

Table S3: Seed sequences for the superfamily of FAD-linked reductases.

| Family name | Seed | Organism |
| --- | --- | --- |
| Proline oxidase family (IPR015659) | Hydroxyproline dehydrogenase  (A6QQ74) | *Bos taurus* |
|  | Proline dehydrogenase 1  (O32179) | *Bacillus subtilis* |
|  | Proline dehydrogenase 1, mitochondrial (O43272) | *Homo sapiens* |
| Eukaryotic-type methylenetetrahydrofolate reductase  (IPR004621) | Methylenetetrahydrofolate reductase  (O80585) | *Arabidopsis thaliana* |
|  | Methylenetetrahydrofolate reductase  (P46151) | *Saccharomyces cerevisiae* |
|  | Methylenetetrahydrofolate reductase  (P42898) | *Homo sapiens* |
| 5,10-methylenetetrahydrofolate reductase (IPR004620) | 5,10-methylenetetrahydrofolate reductase  (P0AEZ1) | *Escherichia coli* |

Table S4: List of primers used for mutagenesis of jMer and sequencing of the ORF of jMer in pT7-7_jMer. The degenerated nucleotides are marked in red.

| Primer name | Sequence (5‘ → 3‘) | Purpose | Annealing temperature |
| --- | --- | --- | --- |
| jMer_E6Q _F | ATTTGGTATCGCATTTGTTCCAAAC | Glu 6 Gln exchange | 59 °C |
| jMer_E6Q _R | TTCATATGTATATCTCCTTCTTAAAG |  |  |
| jMer_D96N _F | CGGTCCAGGAAATAAGGCTACTT | Asp 96 Asn exchange | 63 °C |
| jMer_D96N _R | ATACCTAAAACAGCTCTTCCTC |  |  |
| jMer_N178A_F | AGTTTTAATTGCTGCATCAAACCCAAAAG | Asn 178 Ala exchange | 58 °C |
| jMer_N178A_R | CCATCAGCAATCATACCAG |  |  |
| jMer_N178D_F | AGTTTTAATTGATGCATCAAACCC | Asn 178 Asp exchange | 58 °C |
| jMer_N178D_R | CCATCAGCAATCATACCAG |  |  |
| jMer_F233A_F | AGTTGTTGCAGCCATCGCAGCAG | Phe 233 Ala exchange | 62 °C |
| jMer_F233A_R | GGAACTGCTGCCTGCTTA |  |  |
| jMer_F233L_F | AGTTGTTGCATTGATCGCAGCAG | Phe 233 Leu exchange | 66 °C |
| jMer_F233L_R | GGAACTGCTGCCTGCTTA |  |  |
| pT7-7_Seq_F | TAATACGACTCACTATAGGG | Sequencing of jMer | Not applicable |
| pT7-7_Seq_R | TTGATACCCTTCCTCAGAA |  |  |


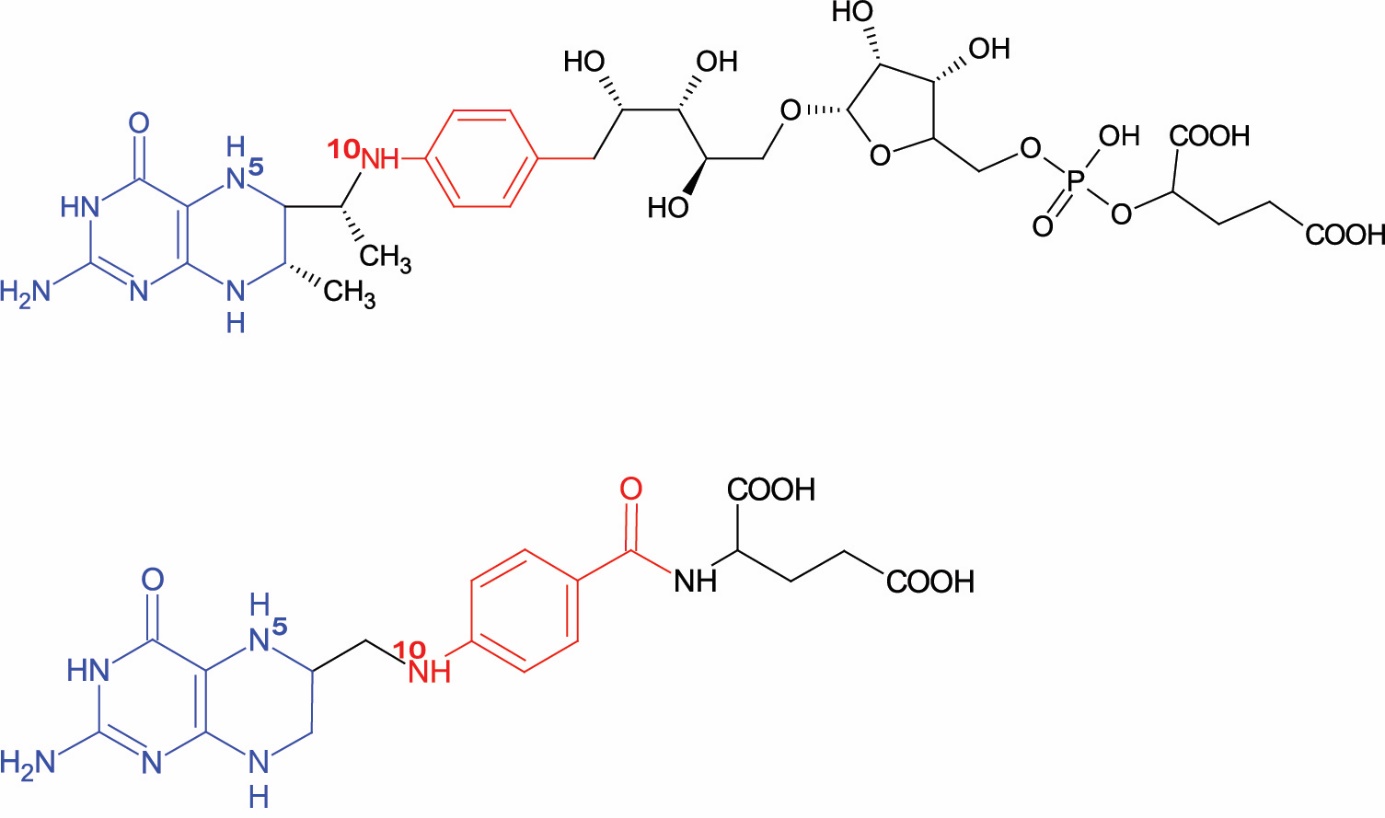


Figure S1: Structures of H_4_MPT (top) and H_4_F (bottom). The pterin part is colored blue. The p-aminobenzoate (PABA) ring of H_4_F and the aniline ring of H_4_MPT are colored red. The tail regions are colored black.


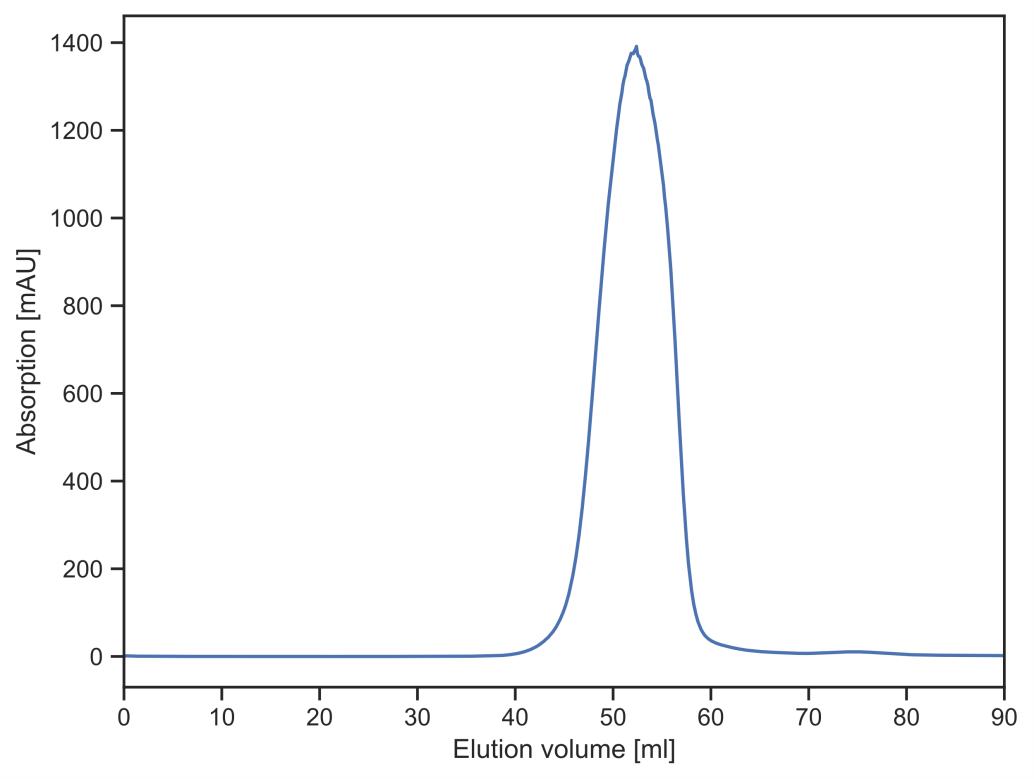


Figure S2: Size-exclusion chromatography of jMer using a HiPrep Sephacryl S-200 HR column. The peak of jMer is centered at around 50 ml elution volume and corresponds to a molecular mass of approximately 80 kDa.


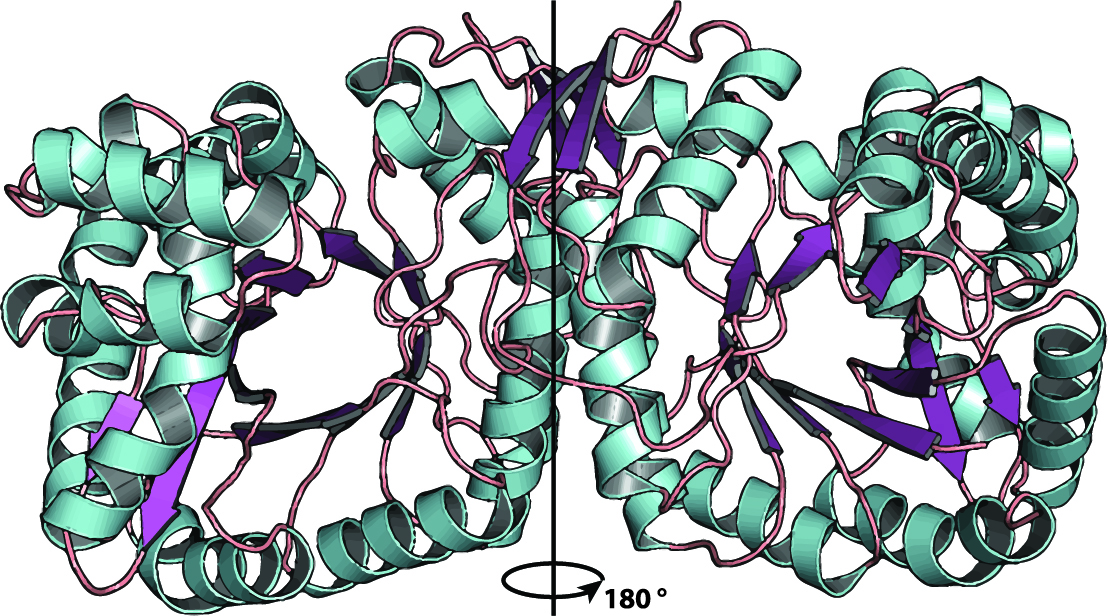


Figure S3: Dimeric structure of jMer. The homodimer is the physiological form of jMer and is formed by a two-fold rotational axis at the center of the protein-protein interface. The active site is located at the C-terminal end of the parallel β-strands, which are positioned on the opposite site of the two monomers.


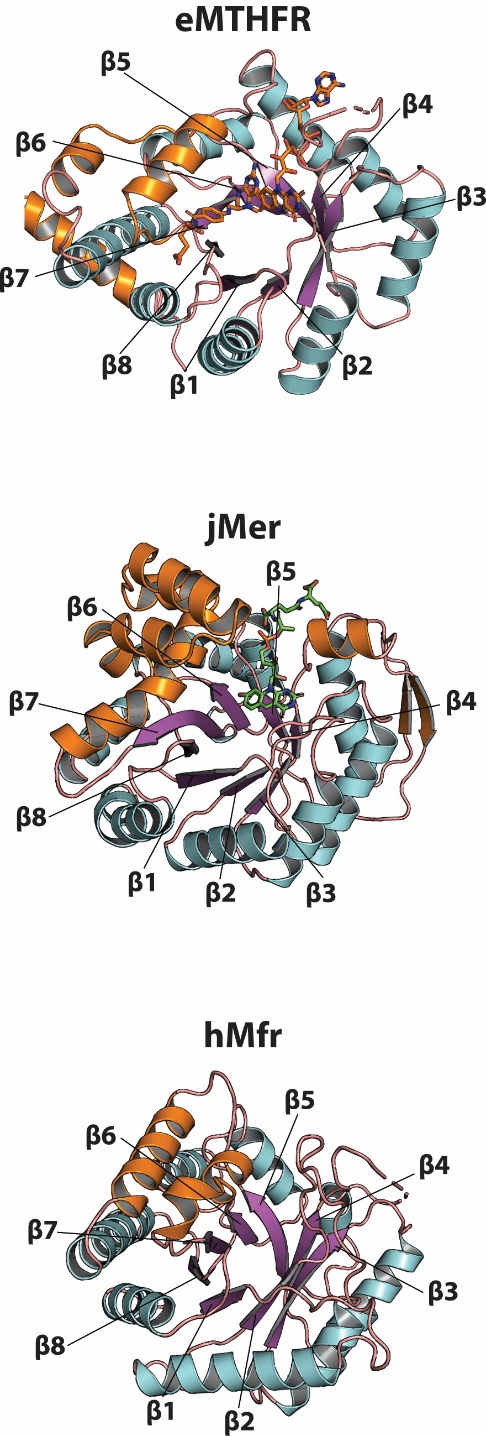


Figure S4: Comparison of the tertiary structures of eMTHFR, jMer and hMfr. The β-strands of the core unit are labeled and colored purple, while the α-helices of the core unit and the loops are colored in light blue and salmon. The inserted helical segment is painted in orange. Methyl-H_4_F and FAD are shown in orange and F_420_ is shown in green.


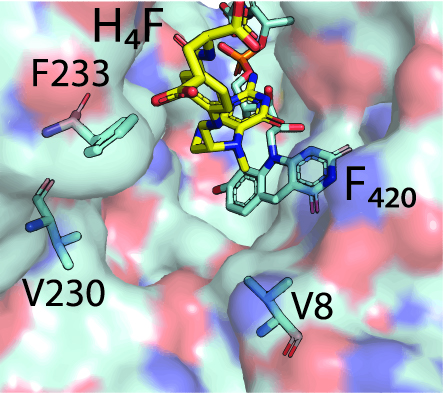


Figure S5: Hydrophobic pocked in jMer. The modeled H_4_F is colored yellow while the native F_420_ and the amino acids are shown in light blue.


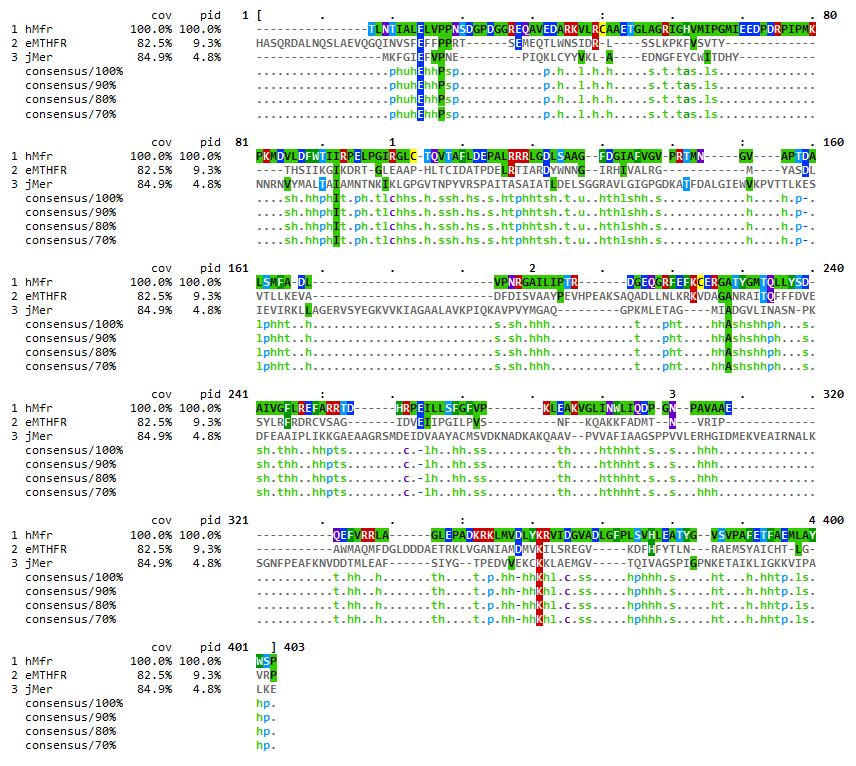


Figure S6: Structure-based alignment of hMfr, eMTHFR and jMer.


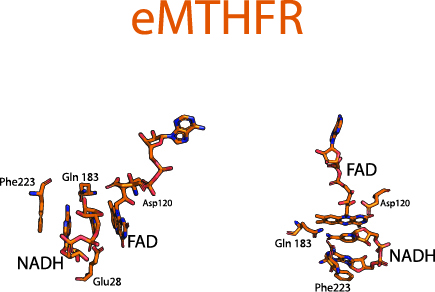


Figure S7: NADH binding site of eMTHFR.


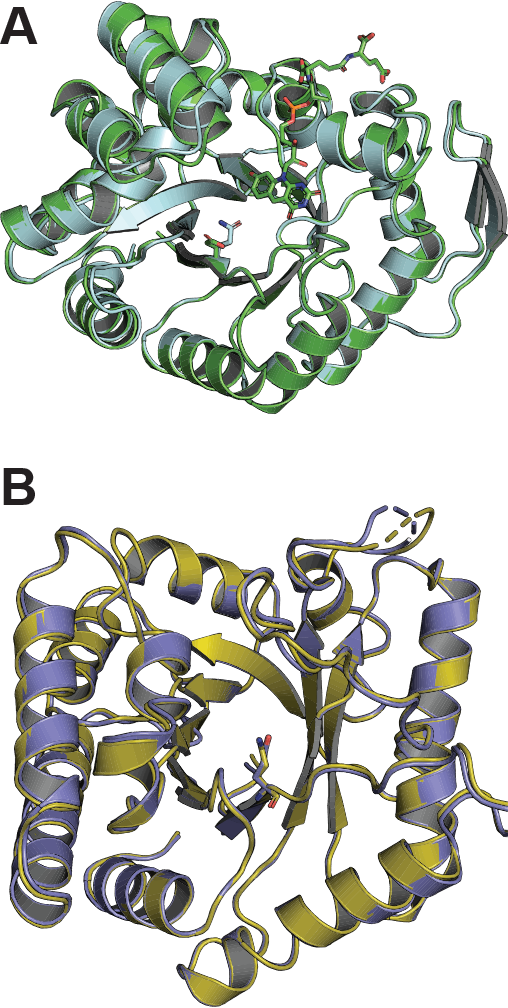


Figure S8: **(A)** Comparison of the structures of jMer wild type (green) and jMer_E6Q (light blue). **(B)** Comparison of the structures of hMfr wild type (dark blue) and hMfr_E9Q (yellow). The glutamate residues are depicted as ball-and-stick model.
