# Supplementary figures and images for: Mutational and structural studies of (βα)8‐barrel fold methylene‐tetrahydropterin reductases utilizing a common catalytic mechanism
Source: Protein Sci. 2024 May 15;33(6):e5018. doi: 10.1002/pro.5018 (PMC11094777; doi:10.1002/pro.5018)

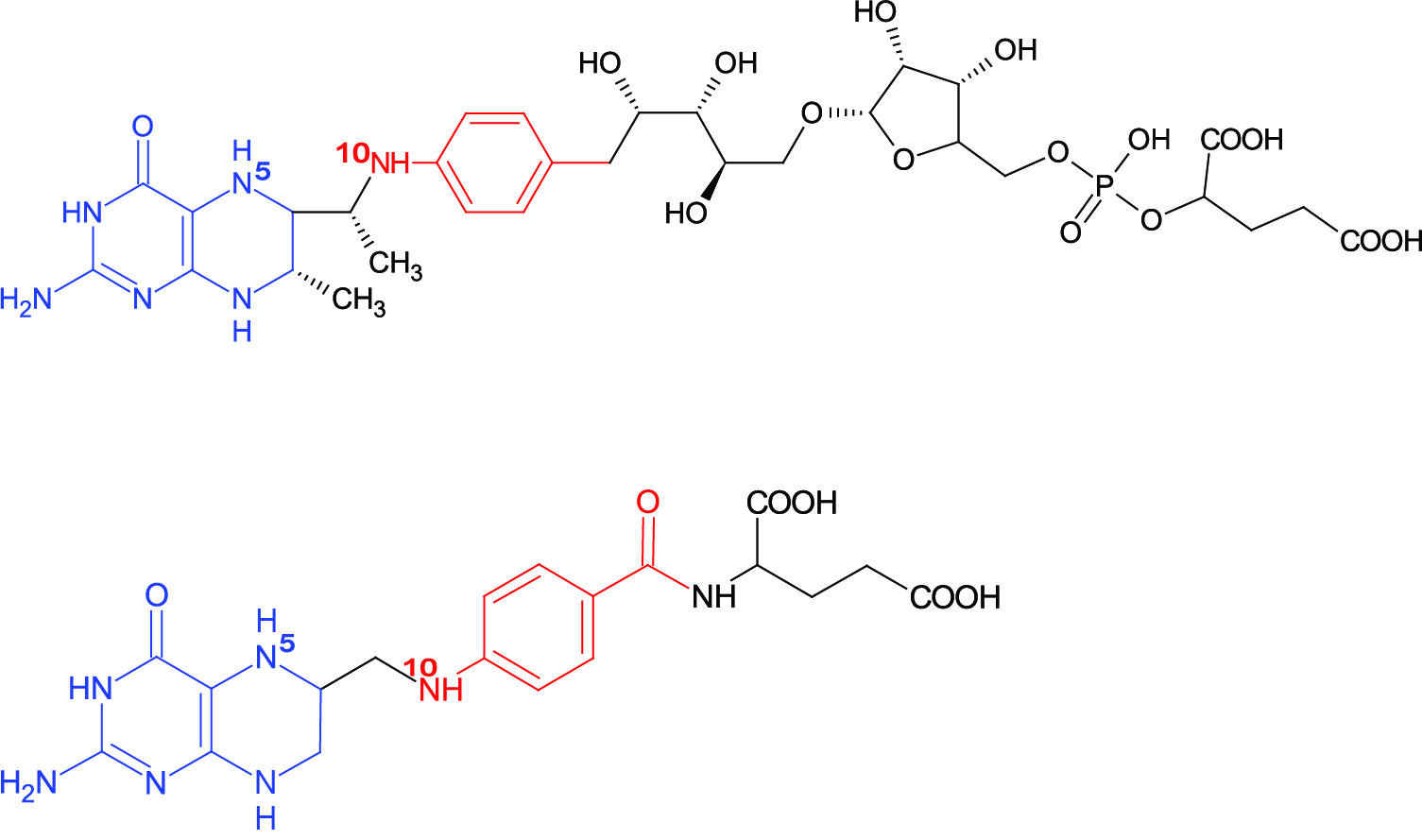

Supplement: Supplementary file 2 — FIGURE S1: Structures of H4MPT (top) and H4F (bottom). The pterin part is colored blue. The p‐aminobenzoate (PABA) ring of H4F and the aniline ring of H4MPT are colored red. The tail regions are colored black. [file PRO-33-e5018-s005.jpg]

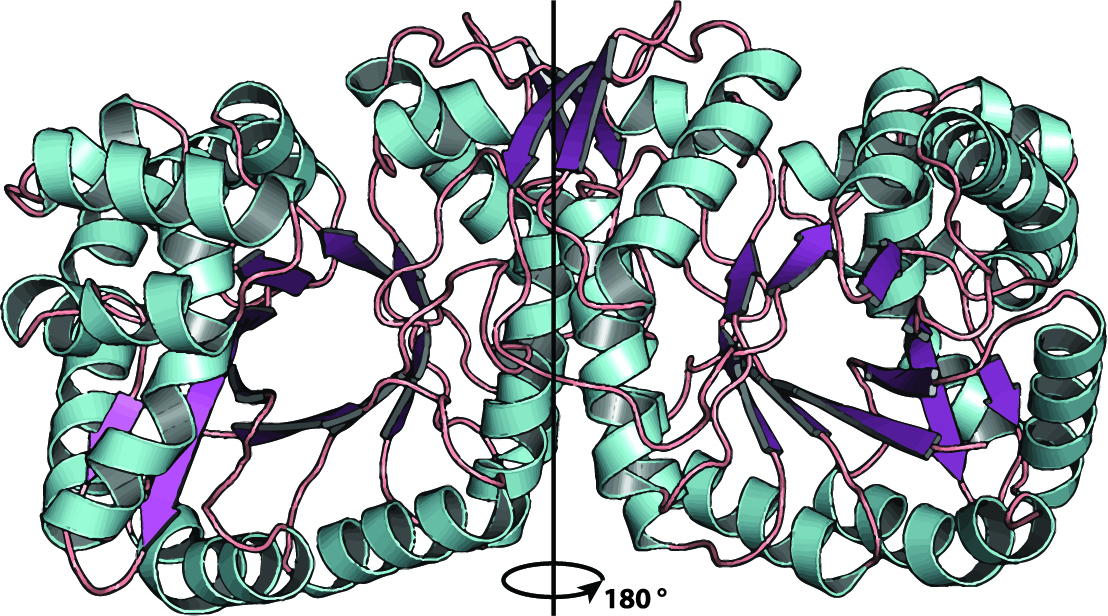

Supplement: Supplementary file 3 — FIGURE S3: Dimeric structure of jMer. The homodimer is the physiological form of jMer and is formed by a two‐fold rotational axis at the center of the protein‐protein interface. The active site is located at the C‐terminal end of the parallel β‐strands, which are positioned on the opposite site of the two monomers. [file PRO-33-e5018-s003.jpg]

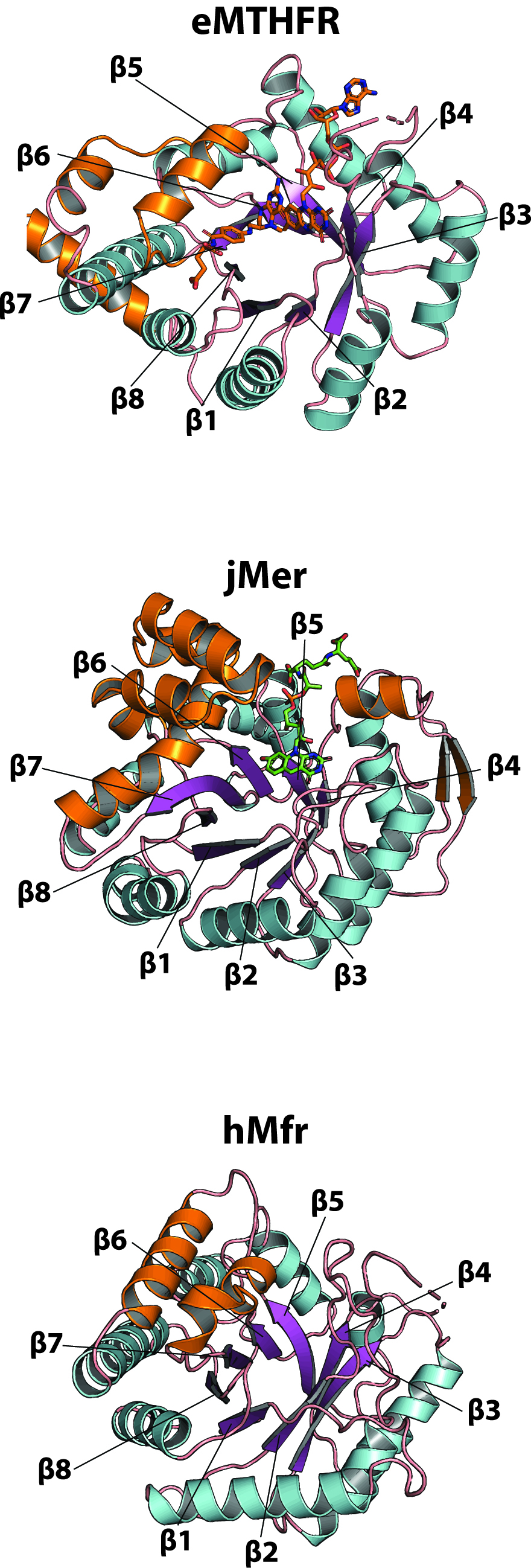

Supplement: Supplementary file 4 — FIGURE S4: Comparison of the tertiary structures of eMTHFR, jMer and hMfr. The β‐strands of the core unit are labeled and colored purple, while the α‐helices of the core unit and the loops are colored in light blue and salmon. The inserted helical segment is painted in orange. Methyl‐H4F and FAD are shown in orange and F420 is shown in green. [file PRO-33-e5018-s002.jpg]

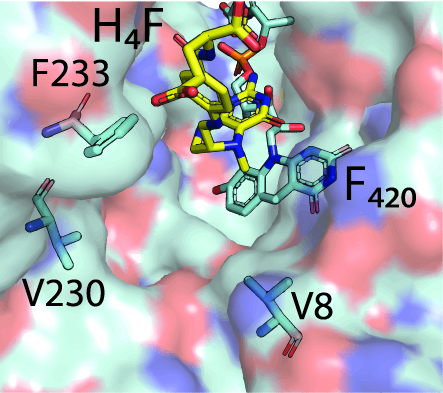

Supplement: Supplementary file 5 — FIGURE S5: Hydrophobic pocked in jMer. The modeled H4F is colored yellow while the native F420 and the amino acids are shown in light blue. [file PRO-33-e5018-s007.jpg]

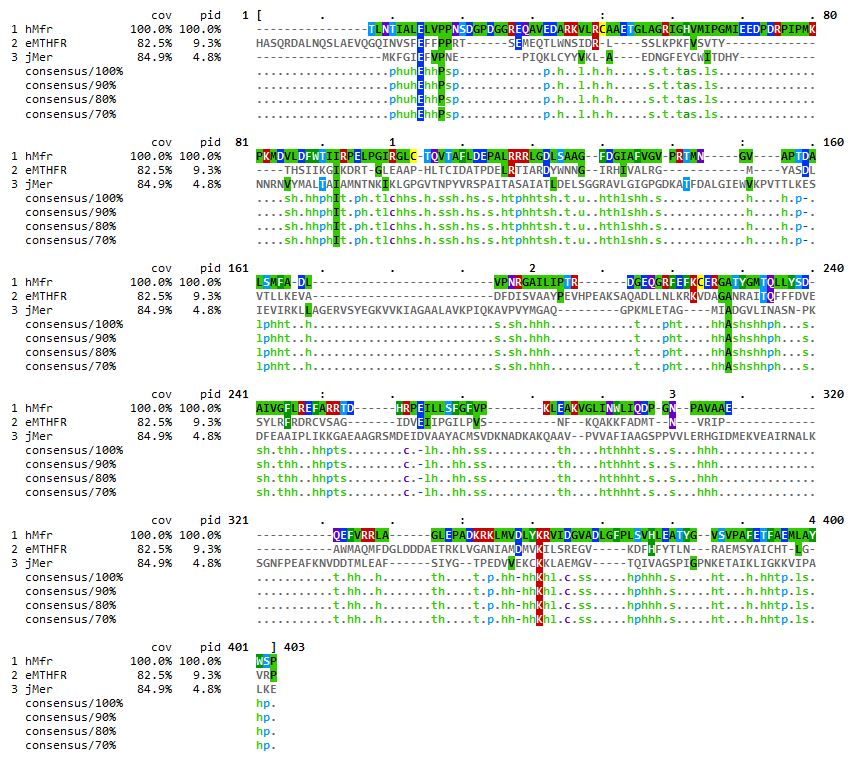

Supplement: Supplementary file 6 — FIGURE S6: Structure‐based alignment of hMfr, eMTHFR and jMer. [file PRO-33-e5018-s008.jpg]

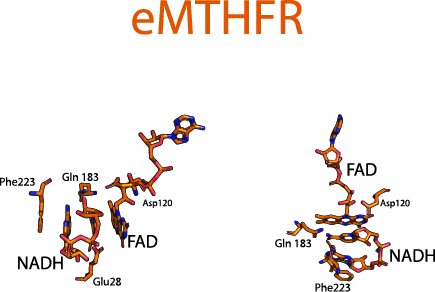

Supplement: Supplementary file 7 — FIGURE S7: NADH binding site of eMTHFR. [file PRO-33-e5018-s006.jpg]

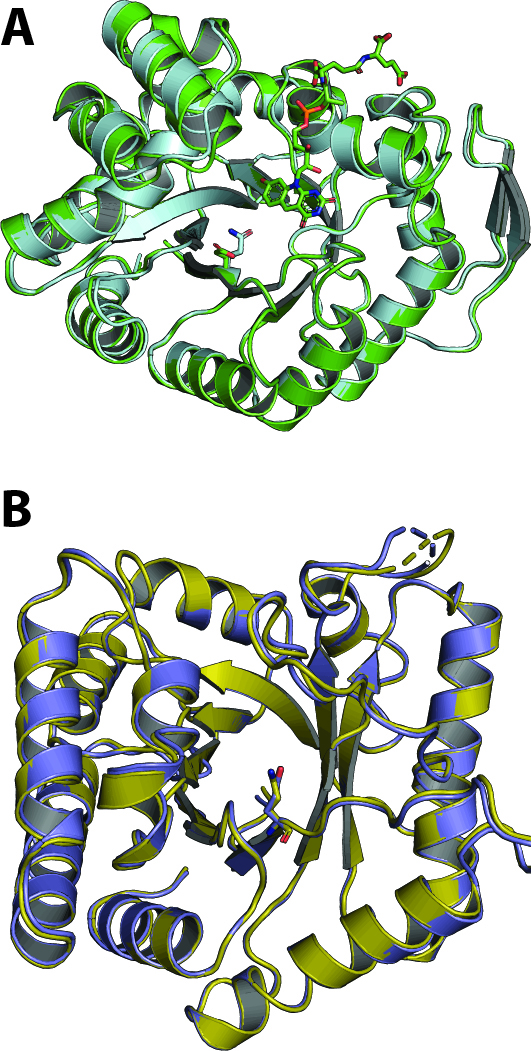

Supplement: Supplementary file 8 — FIGURE S8: (a) Comparison of the structures of jMer wild type (green) and jMer_E6Q (light blue). (b) Comparison of the structures of hMfr wild type (dark blue) and hMfr_E9Q (yellow). The glutamate residues are depicted as ball‐and‐stick model. [file PRO-33-e5018-s004.jpg]
